# Changes in the geographic distribution of the blacklegged tick, *Ixodes scapularis*, in the United States

**DOI:** 10.1016/j.ttbdis.2023.102233

**Published:** 2023-07-24

**Authors:** Lars Eisen, Rebecca J. Eisen

**Affiliations:** Division of Vector-Borne Diseases, National Center for Emerging and Zoonotic Infectious Diseases, Centers for Disease Control and Prevention, 3156 Rampart Road, Fort Collins, CO 80521, United States

**Keywords:** *Ixodes scapularis*, Geographic distribution, Expansion, United States

## Abstract

*Ixodes scapularis* (the blacklegged tick) was considered a species of no medical concern until the mid-1970s. By that time, the tick’s geographic distribution was thought to be mainly in the southeastern United States (US), with additional localized populations along the Eastern Seaboard north to southern Massachusetts and in the Upper Midwest. Since 1975, *I. scapularis* has been implicated as a vector of seven human pathogens and is now widely distributed across the eastern US up to the border with Canada. Geographic expansion of tick-borne diseases associated with *I. scapularis* (e.g., Lyme disease, anaplasmosis, and babesiosis) is attributed to an expanding range of the tick. However, due to changes in tick surveillance efforts over time, it is difficult to differentiate between range expansion and increased recognition of already established tick populations. We provide a history of the documented occurrence of *I. scapularis* in the US from its description in 1821 to present, emphasizing studies that provide evidence of expansion of the geographic distribution of the tick. Deforestation and decimation of the white-tailed deer (*Odocoileus virginianus*), the primary reproductive host for *I. scapularis* adults, during the 1800s presumably led to the tick disappearing from large areas of the eastern US where it previously had been established. Subsequent reforestation and deer population recovery, together with recent climate warming, contributed to *I. scapularis* proliferating in and spreading from refugia where it had persisted into the early 1900s. From documented tick collection records, it appears *I. scapularis* was present in numerous locations in the southern part of the eastern US in the early 1900s, whereas in the north it likely was limited to a small number of refugia sites during that time period. There is clear evidence for established populations of *I. scapularis* in coastal New York and Massachusetts by 1950, and in northwestern Wisconsin by the late 1960s. While recognizing that surveillance for *I. scapularis* increased dramatically from the 1980s onward, we describe multiple instances of clearly documented expansion of the tick’s geographic distribution in the Northeast, Upper Midwest, and Ohio Valley regions from the 1980s to present. Spread and local population increase of *I. scapularis*, together with documentation of *Borrelia burgdorferi* sensu stricto in host-seeking ticks, was universally followed by increases in Lyme disease cases in these areas. Southward expansion of northern populations of *I. scapularis*, for which the host questing behavior of the nymphal stage leads to substantially higher risk of human bites compared with southern populations, into Virginia and North Carolina also was followed by rising numbers of Lyme disease cases. Ongoing surveillance of ticks and tick-borne pathogens is essential to provide the data needed for studies that seek to evaluate the relative roles of land cover, tick hosts, and climate in explaining and predicting geographic expansion of ticks and tick-borne diseases.

## 1. Introduction

*Ixodes scapularis* (the blacklegged tick) has an extensive geographic distribution, including highly diverse environmental conditions, in eastern North America, ranging from Mexico in the south through the United States (US) and into Canada in the north (Guzmán-Cornejo et al., 2007; Guzmán-Cornejo and Robbins, 2010; Ogden et al., 2014; Eisen et al., 2016a; Lindquist et al., 2016). This tick species occurs widely in forested areas of the eastern US, from Florida in the south to Maine in the north along the Eastern Seaboard and extending as far west as the edge of the Great Plains, from Texas in the south to North Dakota in the north (CDC, 2023a,b). Over the last century, the perception of *I. scapularis* has shifted from that of a nuisance tick that occasionally bites humans to the preeminent tick vector of human pathogens in the eastern US. Hooker et al. (1912) noted in their monograph on the life history and bionomics of North American tick species that *I. scapularis* is not known to be associated with pathogen transmission or to be of any great economic importance, but can be found infesting dogs, sheep, and cattle. In similar works from the 1940s, *I. scapularis* was described to occasionally infest humans but still without any known association with transmission of disease agents (Cooley and Kohls, 1945; Bishopp and Trembley, 1945). Since then, more than 150,000 *I. scapularis* ticks have been recorded to infest humans in the US (reviewed by Eisen, 2022), and this tick species has been incriminated as a vector of seven human pathogens: Powassan virus (including the deer tick virus subtype) causing encephalitis; bacterial agents, including *Anaplasma phagocytophilum* causing anaplasmosis, *Borrelia burgdorferi* sensu stricto (s.s.) and *Borrelia mayonii* causing Lyme disease, *Borrelia miyamotoi* causing hard tick relapsing fever, and *Ehrlichia muris eauclairensis* causing ehrlichiosis; and the parasite *Babesia microti* causing babesiosis (Eisen and Eisen, 2018). The majority of vector-borne disease cases reported annually in the United States to the National Notifiable Diseases Surveillance System (NNDSS) are tick-borne; the most commonly reported tick-borne diseases are caused by pathogens spread by *I. scapularis* (NNDSS, 2023).

Field research specifically targeting *I. scapularis* was limited until the discovery in the mid- to late 1970s that this species is a vector to humans of *Ba. microti* in Massachusetts (Spielman, 1976), and the suspicion of an association of bites by *I. scapularis* with cases of erythema chronicum migrans and Lyme arthritis in Connecticut (Steere et al., 1978). These concerns resulted in extensive studies on the occurrence and life history of the tick in coastal areas of the Northeast in the late 1970s (Wallis et al., 1978; Piesman and Spielman, 1979; Piesman et al., 1979; Spielman et al., 1979; Magnarelli et al., 1979; Anderson and Magnarelli, 1980; Carey et al., 1980, 1981; Main et al., 1981, 1982; Coan and Stiller, 1986). The subsequent incrimination in the early 1980s of *I. scapularis* as a vector of the Lyme disease agent now known as *Bo. burgdorferi* s.s. (Burgdorfer et al., 1982) set off a veritable avalanche of field studies on this tick and its associated pathogens across the eastern US. Here, we aim to provide a history of the documented occurrence of *I. scapularis* (including the synonyms *Ixodes dammini* and *Ixodes ozarkus*) in the US from the 1800s to present.

For information about the controversy surrounding the nomenclature of *I. dammini* for northern populations of *I. scapularis*, we refer to the following publications: Spielman et al., 1979; Oliver et al., 1993; Wesson et al., 1993; Caporale et al., 1995; Rich et al., 1995; Keirans et al., 1996; Sanders, 1998; Telford, 1998; Kelly et al., 2014; and Xu et al., 2020. Despite now being considered to belong to the same species, there are important differences in questing behavior and host associations between northern and southern populations of *I. scapularis*. Based on field observations as well as experimental field trials, nymphs of northern populations are prone to quest for hosts more openly, including to ascend vegetation, compared to nymphs of southern populations (Arsnoe et al., 2015, 2019; Tietjen et al., 2020). This difference in questing behavior results in far greater risk of human bites by nymphs from northern populations, which is of direct public health importance as most human infections with *I. scapularis*-associated pathogens in the Northeast and Upper Midwest are considered to be associated with bites by nymphal rather than adult ticks (see Eisen and Eisen, 2016). Moreover, immatures of northern population *I. scapularis* more commonly feed on mammals serving as effective reservoirs for human disease agents, including Lyme disease spirochetes, compared to southern population immatures, which more frequently infest lizards (Ginsberg et al., 2021, 2022). Consequently, pathogen infection prevalence for questing nymphs and adults of *I. scapularis* is generally much higher for northern compared to southern population ticks (Ginsberg et al., 2021; Lehane et al., 2021).

**Fig. 1.**
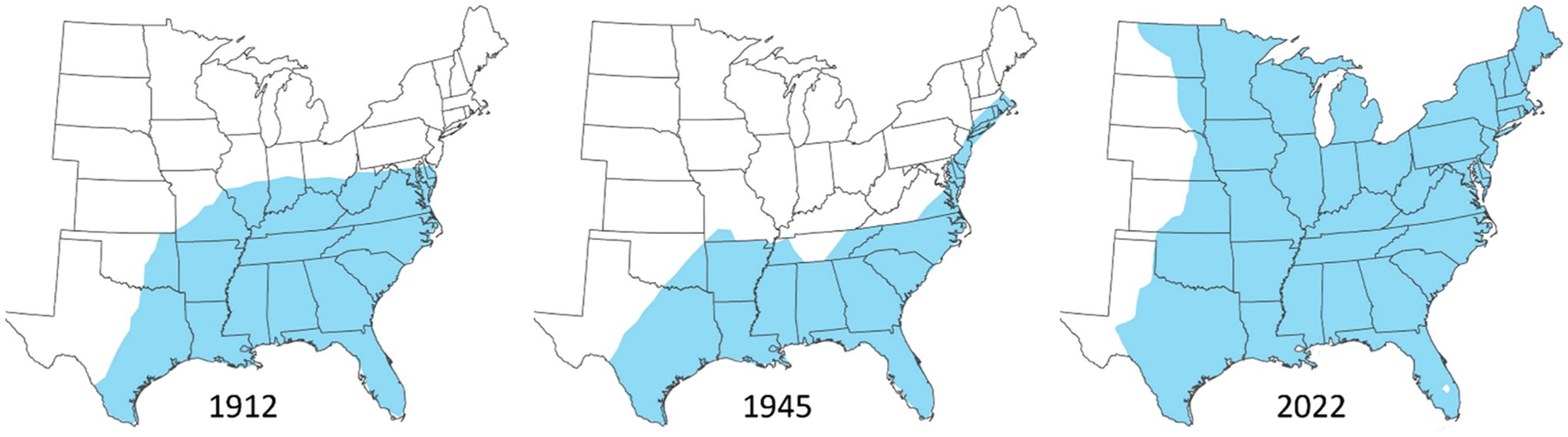
Estimated geographic ranges for *Ixodes scapularis* in the United States based on knowledge of tick collection records by 1912 (Hooker et al., 1912), 1945 (Bishopp and Trembley, 1945), and 2022 (CDC, 2023a).

**Fig. 2.**
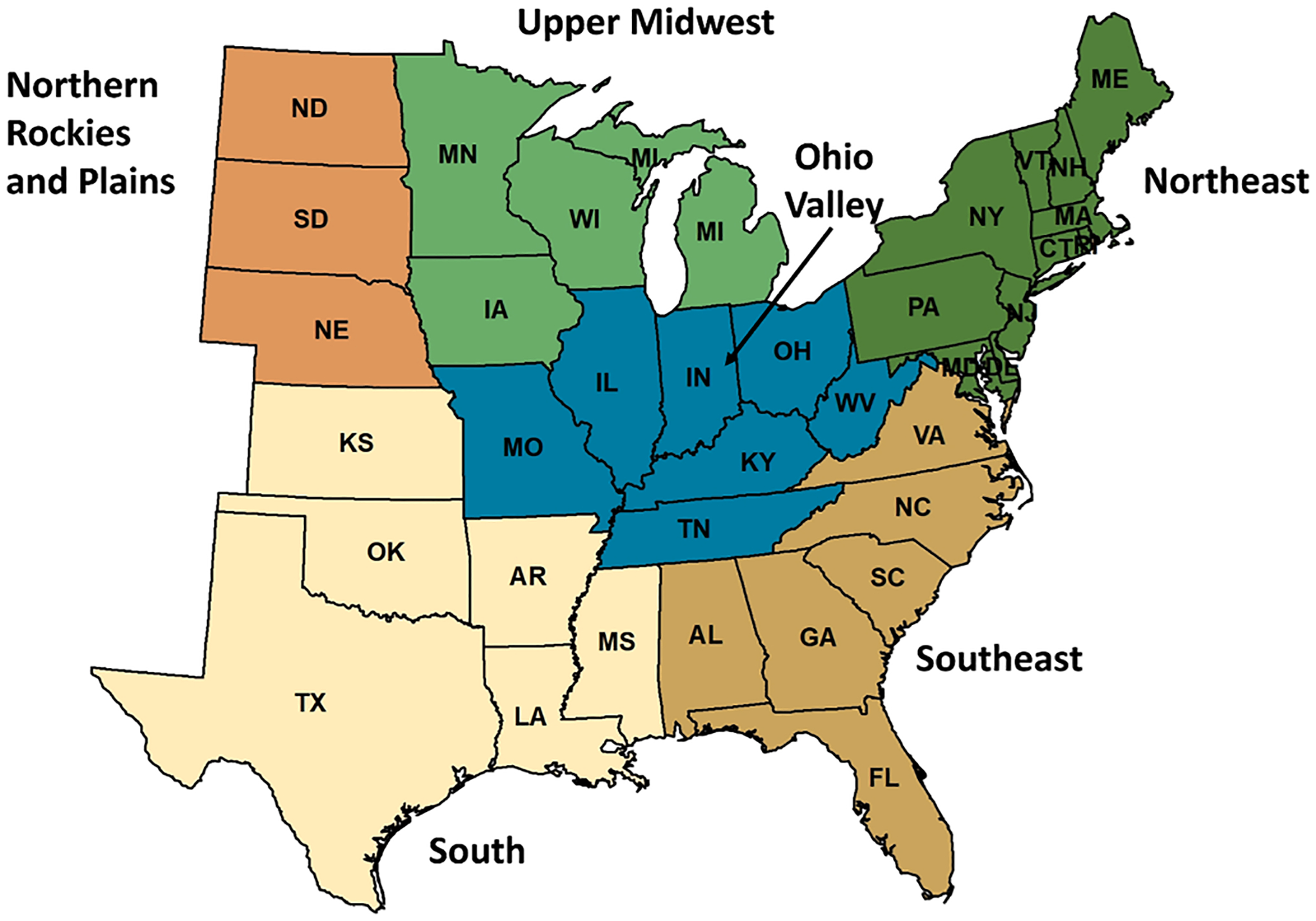
Climate regions of the eastern United States, as defined by the National Oceanic and Atmospheric Administration (NOAA, 2023). The geographic distribution of *Ixodes scapularis* includes the Northeast (Connecticut [CT], Delaware [DE], Maine [ME], Maryland [MD], Massachusetts [MA], New Hampshire [NH], New Jersey [NJ], New York [NY], Pennsylvania [PA], Rhode Island [RI], and Vermont [VT]); the Upper Midwest (Iowa [IA], Michigan [MI], Minnesota [MN], and Wisconsin [WI]); the eastern part of the Northern Rockies and Plains (Nebraska [NE], North Dakota [ND], and South Dakota [SD]); the Ohio Valley (Illinois [IL], Indiana [IN], Kentucky [KY], Missouri [MO], Ohio [OH], Tennessee [TN], and West Virginia [WV]); the Southeast (Alabama [AL], Florida [FL], Georgia [GA], North Carolina [NC], South Carolina [SC], and Virginia [VA]); and the South (Arkansas [AR], Kansas [KS], Louisiana [LA], Mississippi [MS], Oklahoma [OK], and Texas [TX]).

Although there is no doubt that *I. scapularis* has been recorded over an expanded geographic range in the northern part of the eastern US over the last half century (Sonenshine, 2018; Eisen and Eisen, 2022; Fish, 2022; Fig. 1), tick surveillance efforts have also increased, making it difficult to discern how much of this change is attributable to true range expansion versus improved detection and documentation. We have examined the published literature to disentangle examples of true change in the geographic distribution from change that potentially resulted, in part, from increased tick surveillance efforts. Additional detailed records for *I. scapularis* (including records for *Ixodes ricinus scapularis* from the Bishopp collection) came from the United States National Tick Collection (USNTC), housed at Georgia Southern University, Statesboro, GA, USA (USNTC, 2023). These records are referred to in the text as: USNTC, unpublished data. The presentation of tick collection records (Sections 5–11) is broken down by Climate Regions, as defined by the National Oceanic and Atmospheric Administration (NOAA, 2023): (i) Northeast (Connecticut, Delaware, Maine, Maryland, Massachusetts, New Hampshire, New Jersey, New York, Pennsylvania, Rhode Island, and Vermont); (ii) Upper Midwest (Iowa, Michigan, Minnesota, and Wisconsin); (iii) Northern Rockies and Plains, limited to three states (Nebraska, North Dakota, and South Dakota) in the eastern part of the region with records of *I. scapularis*; (iv) Ohio Valley (Illinois, Indiana, Kentucky, Missouri, Ohio, Tennessee, and West Virginia); (v) Southeast (Alabama, Florida, Georgia, North Carolina, South Carolina, and Virginia); and (vi) South (Arkansas, Kansas, Louisiana, Mississippi, Oklahoma, and Texas) (Fig. 2).

## 2. Origin and historical geographic distribution of *I. scapularis*

Genetically based analysis suggests that *I. scapularis* has been present in what is now the US for at least 500,000 years, arising as a species in the southeast with repeated northern expansions interrupted by periods of glaciation (Norris et al., 1996; Van Zee et al., 2015). Following the most recent glaciation of the northern part of the US, roughly 20,000 years ago, *I. scapularis* presumably expanded northward from the southeast at a pace determined by warming climate, reforestation, and northward expansion of vertebrate animals serving as hosts for the immature life stages (a wide range of species including mammals ranging in size from rodents and shrews to ungulates as well as birds and lizards) and the adult stage (predominantly medium-sized and large mammals). Phylogeographic studies indicate that *I. scapularis* first spread from the Southeast to the Northeast, and then from the Northeast to the Upper Midwest (Humphrey et al., 2010). As both *I. scapularis* and *Amblyomma americanum* (the lone star tick) are associated with forested habitats, and the white-tailed deer (*Odocoileus virginianus*) is a key reproductive host for both species, it is reasonable to assume these two notorious human-biting tick species were widely distributed across forested portions of the eastern US prior to the recent European colonization of this part of the country.

## 3. Overview of changes to the geographic distributions of forested land, white-tailed deer, and *I. scapularis* in the eastern US from the start of the European colonization to present

As outlined below, the European colonization of the eastern US apparently led to the disappearance of *I. scapularis* (and *A. americanum*; see Rochlin et al., 2022) from large geographic areas due to deforestation and, especially, decimation of white-tailed deer.

### 3.1. Forested land

The amount of forest cover in the eastern US started to decrease dramatically in the early 1800s and this trend continued for the rest of that century (Li et al., 2023). The lowest point of forest coverage tended to be reached earlier in the Northeast compared to the Upper Midwest; the minimum forest coverage was observed around 1880 in Massachusetts in the Northeast (Foster, 1992; Hall et al., 2002) versus closer to 1930 in Wisconsin in the Upper Midwest (Rhemtulla et al., 2009). These low points were followed by successive reforestation across the eastern US, although the present total forested area is still less than prior to 1800 and there have been changes to forest composition as well as the level of forest fragmentation in some areas (Thompson et al., 2013; Diuk-Wasser et al., 2021; Li et al., 2023).

### 3.2. White-tailed deer

Historical estimates of the population size of white-tailed deer are uncertain but suggest a robust population of 20–30 million individuals in the US and Canada by the late 1500s (McCabe and McCabe, 1984; Adams and Hamilton, 2011). Hunting pressure by both Native North Americans and European colonists led to numbers of white-tailed deer declining in the 1600s and this continued until deer populations reached their lowest point, estimated at 300,000 to 500,000 individuals across the US and Canada, around 1890–1900 (McCabe and McCabe, 1984, 1997; Adams and Hamilton, 2011). This decline in deer numbers was undoubtedly exacerbated by the extensive forest loss in the 1800s. By the late 1800s to early 1900s, white-tailed deer were scarce to absent across most of the Northeast, Upper Midwest, and Ohio Valley regions (Bowles, 1970; Nixon, 1970; Trefethen 1970; Barber, 1984; Blouch, 1984; Gladfelter, 1984; Mattfeld, 1984; Schrauder, 1984; Torgerson and Porath, 1984; McDonald and Miller, 2004). Deer populations did persist in isolated local refugia, such as on Naushon Island off the Massachusetts coast or Long Island in New York, due to landowner management of the deer population (Severinghaus and Brown, 1956; Busby et al., 2008) or in forested tracts in areas that were very sparsely populated by or mostly inaccessible to humans, including in Wisconsin and the central Adirondacks of New York (Dahlberg and Guettinger, 1956; Severinghaus and Brown, 1956; Nixon, 1970). The white-tailed deer appears to have been less severely impacted in some areas of the Southwest and South by the early 1900s, with the greatest numbers of deer thought to remain in parts of Florida, Louisiana, South Carolina, North Carolina, and Virginia (Blackard, 1971; Hanberry and Hanberry, 2020). Remnant deer populations likely persisted in river swamps and rugged mountainous areas. The potential role of alternative hosts for *I. scapularis* adults (such as medium-sized mammals, cattle, or turkeys) to maintain local tick populations as numbers of white-tailed deer declined is not clear for either the southern or northern parts of the eastern US.

Over the 1900s, the combination of successive reforestation of the eastern US (Li et al., 2023), legislation to protect white-tailed deer (Trefethen, 1970; Adams and Hamilton, 2011), and deer restocking and population management (McDonald and Miller, 2004; Diefenbach and Shea, 2011; VerCauteren and Hygnstrom, 2011) led to dramatic increases in white-tailed deer populations (McCabe and McCabe, 1997; Adams and Hamilton, 2011; Hanberry and Hanberry, 2020). White-tailed deer were restocked in most states of the eastern US, with the exception of Connecticut, Delaware, Maine, Massachusetts, Michigan, Minnesota, New Hampshire, and Wisconsin (reviewed by McDonald and Miller, 2004). Deer restocking started already in the late 1800s in a few states (Iowa, North Carolina, New York, and Vermont) and deer restoration efforts were completed by the early 1970s in the majority of eastern states. The restocking efforts in most cases primarily included within-state translocation of deer, but many states also restocked smaller numbers of deer from out-of-state source locations (McDonald and Miller, 2004). Notably, Wisconsin was a primary source for out-of-state deer restocking, with animals translocated both to northern and southern states (Alabama, Arkansas, Florida, Georgia, Indiana, Kentucky, Louisiana, Mississippi, North Carolina, Tennessee, West Virginia, and Virginia). Other prominent sources for out-of-state deer restocking included Michigan, North Carolina, Pennsylvania, and Texas. The resurgence of deer was also likely aided by a warming climate from the late 1900s onward, with milder winter temperatures and reduced snow depth (Dyer and Mote, 2006; Contosta et al., 2019) favoring deer survival in the winter months (Matschke, 1984; Mech, 1984; Nelson and Mech, 1986; DelGiudice et al., 2002). By 2000, the number of white-tailed deer in the US and Canada had rebounded to the 25–30 million range, similar to the estimated population size by the late 1500s (Adams and Hamilton, 2011).

### 3.3. Ticks

The deforestation and decimation of deer appears to have resulted in contraction of the geographic distribution, relative to the presumed pre-colonization distribution, for both *I. scapularis* and *A. americanum* during the 1800s and early 1900s. Rochlin et al. (2022) describes this process for *A. americanum*, starting with the first documentation of this species by the naturalist Pehr Kalm from the New Jersey/Pennsylvania area in 1749 and leading up to the ongoing northward expansion of *A. americanum* across the eastern US. It also should be noted that Kalm (1772) described ticks as being abundant in the woods in 1749, whereas roughly a century later Fitch (1872) stated that the previously abundant *A. americanum* (=*Ixodes americanus*) had declined to become nearly extinct in parts of the Northeast due to forest clearing but was still abundant in sparsely settled parts of the country further west and south.

No such clear statement exists in the early literature for *I. scapularis* with regards to changes in its geographic distribution during the 1800s. We nevertheless make the reasonable assumption, supported by population genetics analyses (Humphrey et al., 2010), that *I. scapularis* was widely distributed and abundant in the forests of the eastern US prior to and in the early phases of the European colonization. Based on supportive data presented in Sections 5–11 for the time period from 1821 to 1950, we conclude that: (i) *I. scapularis* had become limited to isolated forested geographic refugia where deer still persisted in the Northeast, Upper Midwest, and Ohio Valley regions by the late 1800s and early 1900s, presumably as a result of deforestation and severe depredation of white-tailed deer; and (ii) *I. scapularis* persisted to a greater extent in the 1800s and early 1900s across the Southeast and South regions, where white-tailed deer still were present in some areas, albeit in reduced numbers. Based on supportive data presented in Sections 6–11 for the time period from 1951 to present, we further conclude that reforestation and population increase and spread of white-tailed deer allowed *I. scapularis* to emerge from local refugia to spread across and proliferate in the Northeast, Upper Midwest, and Ohio Valley regions. It remains unknown to what extent deer translocated in restocking efforts may have been infested with ticks, thus potentially facilitating spread of *I. scapularis* within or between states. The spread and proliferation of *I. scapularis* in the northern part of the eastern US, and into Canada, also likely was facilitated by a warming climate in recent decades, as rising temperatures result in a longer season when the tick can be active (seeking hosts, feeding, and developing to the next life stage or laying eggs) and may favor overwinter survival (Eisen et al., 2016b; Ogden et al., 2021). The early distribution records summarized in this paper, combined with population genetic studies (Qiu et al., 2002; Humphrey et al., 2010; Sakamoto et al., 2014; VanZee et al., 2015; Beati et al., 2023), support the view that *I. scapularis* in large part may have reclaimed a historical range in the eastern US over the last century rather than spread into areas where this tick species had never before been present.

The number and specific locations of refugia where *I. scapularis* was able to persist through the early 1900s remain unclear. However, there are indications from the Northeast that the tick persisted locally in coastal areas stretching from Delaware/New Jersey to southern Massachusetts, and then has spread inland as well as north into Maine/New Hampshire/Vermont, and into Canada (see Section 6). In the Upper Midwest, the tick appears to have persisted locally in northern forests, including in northwestern Wisconsin, and then spread in all cardinal directions across the region and into Canada (see Section 7). Spread across the Ohio Valley region was likely associated with incursions from multiple directions: from the Upper Midwest southward into Indiana and Illinois; from the Northeast into Ohio and West Virginia; and from the south into Missouri, Kentucky, Tennessee, and West Virginia (see Section 9). Spread of *I. scapularis* within the South and Southeast regions was less dramatic, and not as well documented, as the tick may have persisted in a greater number of locations during its decline (see Sections 10–11).

The geographic spread of *I. scapularis* in the eastern US, and in Canada, over the last half-century likely resulted from a combination of shorter-range dispersal via various hosts (including ungulates, carnivores, and non-migratory birds) and longer-range dispersal by migratory birds (Ogden et al., 2008; Tsao et al., 2021). There is extensive documentation of *I. scapularis* infesting hunter-killed white-tailed deer during surveys conducted in the early phases of the tick invading new areas in the Northeast, Upper Midwest, and Ohio Valley regions (see Sections 6.4, 6.7, 7.4, 7.6, 9.4, 9.5, and 9.6). However, the relative importance of deer versus birds for the spread of *I. scapularis* to new areas in the US remains unclear, especially along major migration routes where birds collectively can transport large numbers of ticks (Weisbrod and Johnson, 1989; Rand et al., 1998; Durden et al., 2001; Brinkerhoff et al., 2011).

## 4. Background to the regional summaries of collection records for *I. scapularis* in the eastern US from 1821 to present

Sections 5–11 summarize collection records for *I. scapularis* from the description of the species by Say in 1821 to present. As records from 1821 to 1920 are scarce and generally provided very limited detail about the tick collections, they are presented collectively for the eastern US (Section 5). Collection records from 1821 to 1920 often came from humans, dogs, or livestock, and could potentially be related to tick exposures outside of the state from which they were reported. Despite occasional records from the Northeast, Upper Midwest, and Ohio Valley regions, there appears to be a building consensus in the very early 1900s of *I. scapularis* as a southern tick species. In a notable achievement, Hooker et al. (1912) presented the first map showing an estimated geographic range of *I. scapularis*, extending from the Southeastern Seaboard to central Texas and Oklahoma, and with the northern extent of the range traversing Missouri, Illinois, Indiana, Ohio, West Virginia, Maryland, and Delaware (Fig. 1).

Records from 1921 to present included more information about collection location and year, the source of ticks, and life stages and numbers of ticks recovered. Records from this time period are presented broken down by climate region (Sections 6–11). Records for *I. scapularis* were still scarce from 1921 to 1950, when it was considered a minor nuisance biter without either severe economic impact on livestock or association with human disease. Collection records typically resulted from studies focusing broadly on ectoparasites of wildlife or domestic animals, or on tick species of concern for livestock (*Rhipicephalus* cattle ticks) or human health (the American dog tick, *Dermacentor variabilis*). From 1921 to 1950, collection records typically include small numbers of adult ticks taken from humans or domestic animals, or from hunter-killed medium-sized mammals (e.g., bobcat, coyote, fox, opossum, and raccoon) or deer (see references in Sections 6–11). Records from drag sampling or examination of wildlife serving as hosts for the immature life stages, such as rodents, lizards, and birds, occurred but were less frequent (Larousse et al., 1928; Hixson, 1941; Smith and Cole, 1943; Cooley and Kohls, 1945; Collins et al., 1949; Rogers, 1953; White, 1955; Eads et al., 1956; Spielman et al., 1979; Persing et al., 1990; Mc Allister et al., 2016). Records of *I. scapularis* from 1921 to 1950 were most plentiful and widespread in the Southeast and South climate regions, limited from the Ohio Valley region, entirely lacking from the Upper Midwest and Northern Rockies and Plains, and restricted to local geographic areas in the Northeast. Notable works published in the 1940s (Cooley, 1944; Bequaert, 1945; Bishopp and Trembley, 1945; Cooley and Kohls, 1945; Collins et al., 1949) described *I. scapularis* as a species with a southern distribution. The estimated geographic range of *I. scapularis* (= *I. ricinus scapularis*) presented by Bishopp and Trembley in 1945 depicts a southern distribution for this tick in the eastern US, fairly similar to that presented previously by Hooker et al. (1912) but extending further north along the Eastern Seaboard up to Massachusetts (Fig. 1).

Records for *I. scapularis* remained relatively scarce from 1951 to 1975, as the tick was still considered a minor nuisance biter and most records were from studies focusing broadly on ectoparasites of wildlife or domestic animals, or on other tick species. Similar to the period from 1921 to 1950, collection records typically included small numbers of adult ticks taken from humans or domestic animals, or from hunter-killed mammals (see references in Sections 6–11). This, however, included notable examples of systematic and geographically widespread collection of ticks from hunter-killed deer in the Southeast and South (Kellogg et al., 1971), and in Wisconsin in the Upper Midwest (Jackson and Defoliart, 1970). Records from drag sampling or examination of wildlife serving as hosts for the immature life stages, such as rodents, lizards, and birds, were less frequent and mostly included small numbers of ticks (Eads et al., 1956; Jackson and Defoliart, 1970; Sonenshine and Stout, 1971; Wilson and Baker, 1972; Wilson and Kale, 1972; Spielman, 1976; McEnroe, 1977; Spielman et al., 1979), but in some instances were extensive enough to recover hundreds of immatures from hosts or adults by drag sampling (Tugwell and Lancaster, 1962; Clymer et al., 1970; Good, 1973). Records published in scientific journals or monographs for *I. scapularis* from 1951 to 1975 accumulated at a rate comparable to the 1940s for the Southeast, South, and Ohio Valley regions. In contrast, the first collections of this tick were recorded from the Upper Midwest and Northern Rockies and Plains, and records from the Northeast were increasingly widespread. One additional notable development, related to animal health, was that the United States Department of Agriculture (USDA) started conducting annual national tick surveys in 1963 (USDA, 1964), which continued until 1987 (USDA, 1988). These annual surveys generated records for a wide range of tick species, including *I. scapularis*, collected from cattle, horses and mules, dogs, native wildlife (including medium-sized mammals and deer), and various other hosts (including zoo animals, humans, and domestic animals other than cattle, horse/-mule, and dog). Results from the USDA surveys were similar to data from the other sources up to 1975: the majority of *I. scapularis* were collected in the Southeast and South.

**Fig. 3.**
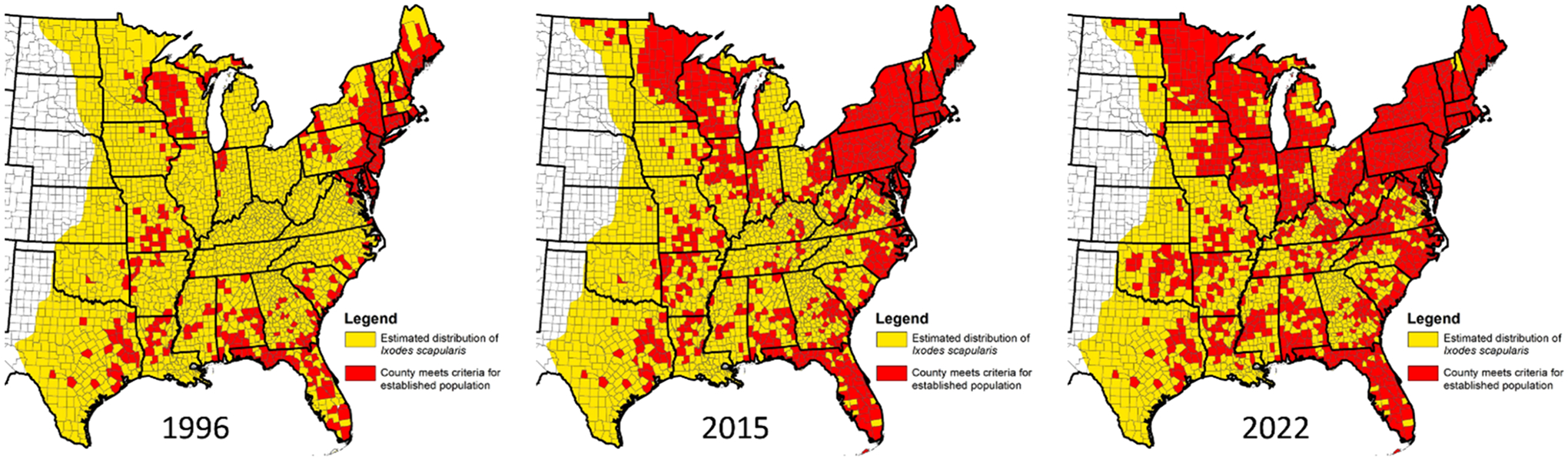
Distribution of counties (shown in red) where *Ixodes scapularis* was classified as established (6 or more ticks of a single life stage or 2 or more life stages recorded) in 1996 (Dennis et al., 1998), 2015 (Eisen et al., 2016a) and 2022 (CDC, 2023b). The underlying estimated range of where the tick can survive and reproduce is shown in yellow (Hahn et al., 2016, 2017).

**Fig. 4.**
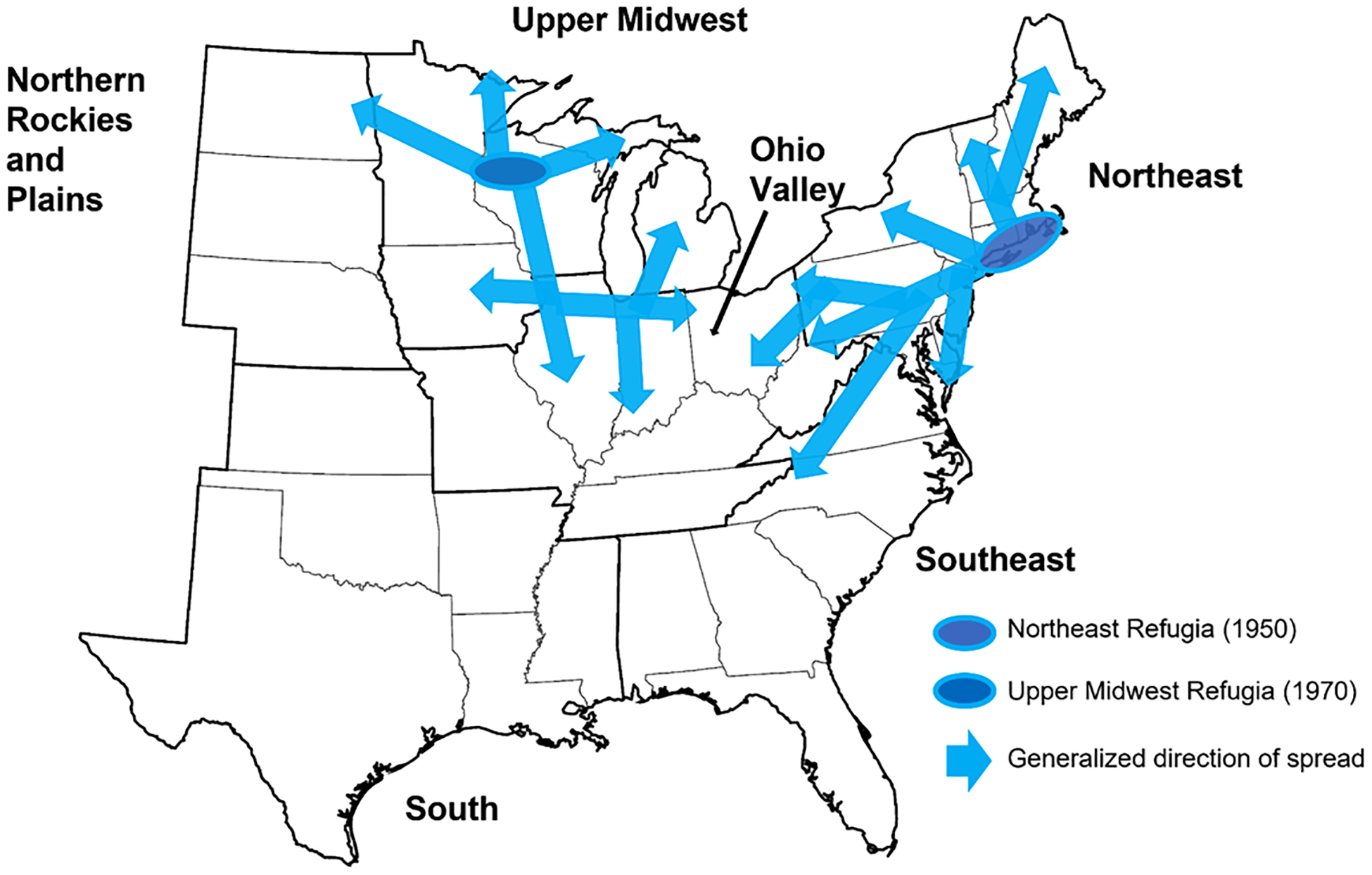
Locations of refugia for northern populations of *Ixodes scapularis*, where the tick was documented to be present and abundant in the Northeast by 1950 (coastal areas from New York to southern Massachusetts) and in the Upper Midwest by 1970 (northwestern Wisconsin). The arrows show presumed directions of spread from these refugia until present, based on data described in Sections 6 to 10.

The realization in the late 1970s and early 1980s that *I. scapularis* is a vector of human pathogens causing babesiosis and Lyme disease resulted in the initiation of numerous field studies across the eastern US that specifically targeted this tick. There also was a notable shift away from humans, domestic animals, and wildlife serving as the primary sources for recovered ticks toward field studies commonly using drag/flag sampling to collect host-seeking ticks. More recently, however, large numbers of *I. scapularis* have also been recorded from humans and pets via passive surveillance programs where ticks are provided to scientists by the public or medical or veterinary practitioners (reviewed by Eisen and Eisen, 2021). There is no doubt that increased surveillance efforts have resulted in improved documentation of the geographic distribution of counties with established populations of *I. scapularis* from 1976 to present (Fig. 3). However, as outlined in Sections 6–9 and illustrated in Fig. 4, there are also well documented examples of geographic spread of *I. scapularis* during this time period within the Northeast and Upper Midwest, as well as from these regions into the Ohio Valley region. For the period from 1976 to present we therefore place special emphasis on data providing clear evidence of geographic spread or population increase of this tick, versus accumulation of tick records based simply on increased surveillance effort.

Dennis et al. (1998) published the first national summary of counties where *I. scapularis* had been reported or was considered to be established (i.e., at least six individuals of a single life stage or at least two life stages detected in the county), based on data up to 1996. Records were based on literature review, a tick mapping questionnaire sent to public health officials, acarologists and Lyme disease researchers, and review of USNTC records. Eisen et al. (2016) updated the national county-level distribution map making a minor modification to the “established” definition used by Dennis et al. (1998); established counties were defined by at least six individuals of a single life stage or at least two life stages detected in the county *within a single year*. County records included those recorded in Dennis et al. (1998), with county status updated based on publications from 1996 through 2015, records from state health department websites, and reports from public health officials, acarologists, and Lyme disease investigators throughout the US. A national tick and tickborne pathogen surveillance program initiated by the Centers for Disease Control and Prevention (CDC) in 2018 (Eisen and Paddock, 2021) also contributed to improved knowledge of where *I. scapularis* is established. As part of that initiative, tick data are collated from state health departments and other public health partners in ArboNET. Definitions in ArboNET of counties where *I. scapularis* is considered reported and established conform with Eisen et al. (2016). The distributions of counties where the tick was considered to be established by 1996 and subsequently by 2015 and 2022 are shown in Fig. 3.

**Fig. 5.**
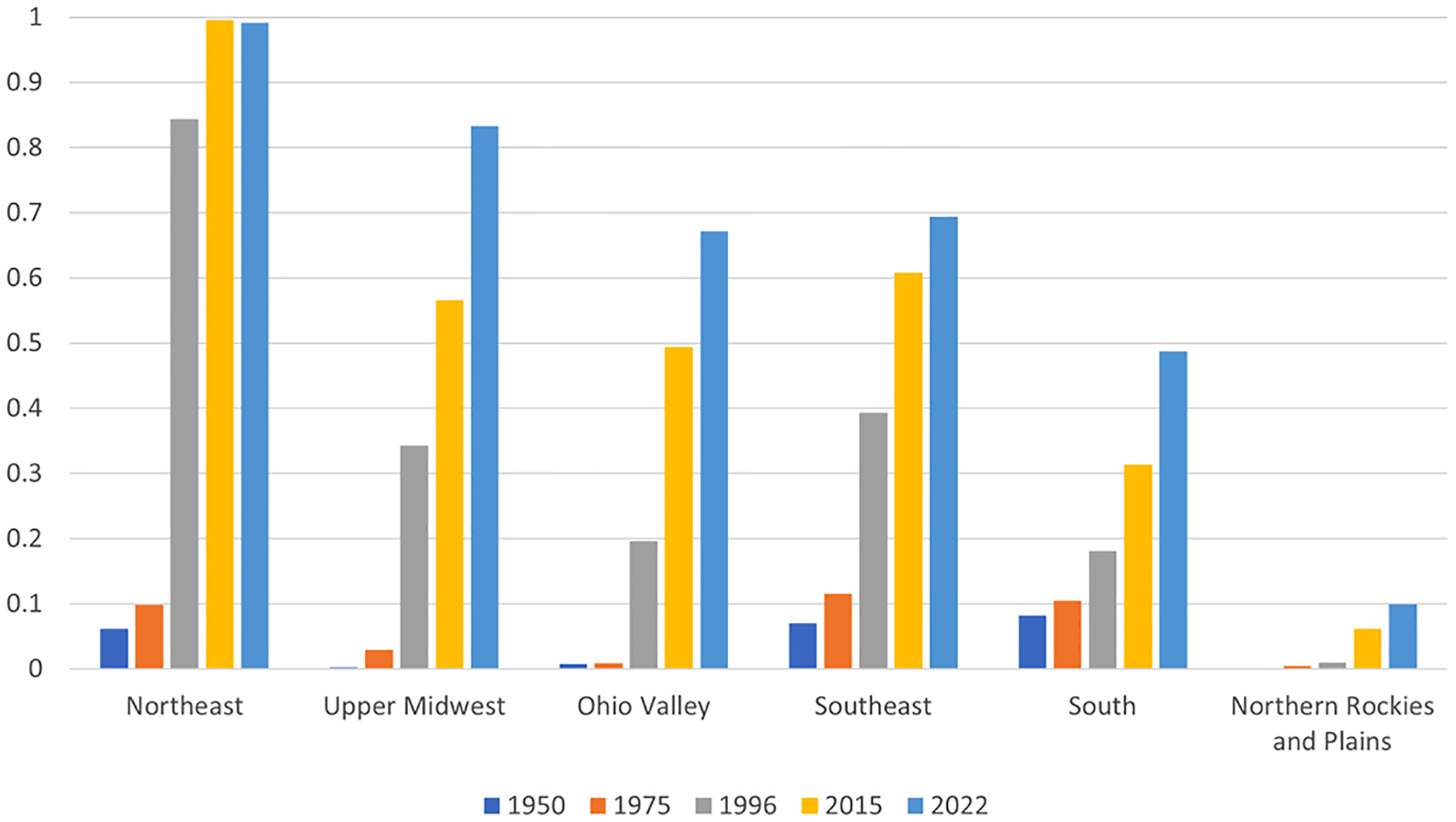
Cumulative proportion of counties by region in the eastern United States with *Ixodes scapularis* reported (i.e., based on collection locations identifiable to county level) up to 1950, 1975, 1996, 2015, and 2022. Only Nebraska, North Dakota, and South Dakota are included for the Northern Rockies and Plains region. Counts of counties are based on information presented in Sections 5–11 for 1950 and 1975, and by Dennis et al. (1998) for 1996, Eisen et al. (2016a) for 2015, and CDC (2023b) for 2022.

The most current estimated geographic range map displays the northern range limit for *I. scapularis* in the eastern US as coinciding with the Canadian border from central North Dakota eastward to Maine on the Atlantic Coast (Fig. 1). As outlined in Sections 6–9, collection records generated from 1976 to present have both moved the range limit northward (Figs. 3–4) and documented the presence of *I. scapularis* from a large number of new counties within the Northeast, Upper Midwest, Northern Rockies and Plains, and Ohio Valley regions (Fig. 5).

The estimated geographic range for *I. scapularis* in the South and Southeast regions by 2022 was much the same as in the 1940s (Fig. 1), although surveillance efforts have documented the presence of this tick from new counties within the South and Southeast over time (Fig. 5). One notable recent development is the southward spread of northern populations of *I. scapularis* from the Northeast into West Virginia, Virginia, and North Carolina (van Zee et al., 2015; see also Sections 9.7 and 10.4–10.5), where previously only southern populations of this species occurred. This is concerning because, as noted in Section 1, nymphs of northern populations are prone to quest for hosts more openly compared to nymphs of southern populations, resulting in far greater risk of human bites and pathogen transmission by nymphs from northern populations.

## 5. Geographic distribution of *I. scapularis* records in the eastern US from 1821 to 1920

*Ixodes scapularis* was first described by Say (1821), without a type locality or a type host but with the following general note: “Rather common in forests, and frequently found attached to different animals.” The next documentation of this species came seven decades later, when Curtice (1892) mentioned *I. scapularis* infesting cattle in New York and noted that this tick commonly infests dogs and small wild animals in the eastern US. However, no further details were provided for specific locations or years of collection. A decade later, Neumann (1901) included records of *I. scapularis* (as *Ixodes ricinus*) from the Southeast (Florida and North Carolina), South (Texas and Kansas), and Northeast (Maryland and Pennsylvania), but without any mention of hosts or specific locations or years of collection. Hunter and Hooker (1907) similarly noted *I. scapularis* from Florida and Texas, again without further information. Banks (1908) then noted that *I. scapularis* occurs in many places in the southernmost part of the eastern US, being especially abundant in Florida and southern Texas, but also mentioned sporadic collections further north along the Atlantic Coast in North Carolina, Virginia, and Maryland, as well as in the Upper Midwest (Iowa) and Ohio Valley region (Indiana). Further details about these collection records were sparse, but in some cases included the source of the ticks (human and dog for Florida and Texas; cattle for Virginia; and sheep for Maryland) or collection location (McGregor in Clayton County for Iowa; the city of Norfolk in Virginia). Hooker et al. (1912) provided new collection records from Florida, Louisiana, Texas, and Tennessee; and mentioned previous collections from Maryland south to Florida along the Eastern Seaboard and in the central states of Missouri, Indiana, and Iowa. Although still not specifying the year of collection, some of the new records did include information for collection location, host, and life stage: adults taken from dog(s) and opossum(s) in Alachua and Orange counties in Florida; and a single female taken from an opossum in Lee County, Texas.

We are aware of only four records for *I. scapularis* in the published literature with a specified year of collection prior to 1920: (i) four females and four males taken from white-tailed deer in Craven County, North Carolina in 1897 (Nuttall et al., 1911); (ii) four females taken from an unknown source in Miami-Dade County, Florida in 1909 (Nuttall et al., 1911); (iii) one female and one male taken from human(s) in Elk County, Pennsylvania in 1912 (Pak et al., 2019); and (iv) an unspecified number of females taken from raccoon(s) in Miami-Dade County, Florida in 1914 (Cooley and Kohls, 1945).

Additional records from the United States National Tick Collection (USNTC, unpublished data) may to some extent overlap with the published records outlined above but do provide more detailed information about the tick collections. Similar to the published literature, the majority of records for *I. scapularis* in the USNTC up to 1920, ranging in collection years from 1897 to 1919, were from the South and Southeast regions (32 and 15 records, respectively). These records are dominated by adult ticks collected from humans or domestic animals. Counties of collection were included for a subset of records from Louisiana (Madison), Mississippi (Franklin, Jefferson, Washington, and Yazoo), and Texas (Austin, Dallas, Kerr, Lee, Marion, Matagorda, and Victoria) in the South region, and for Florida (Alachua, Orange, and Volusia), North Carolina (Cumberland), and South Carolina (Georgetown) in the Southeast region. A few additional records of *I. scapularis* came from Tennessee in the Ohio Valley region: four records including a total of 29 adult ticks collected in 1911 from livestock, including from Fentress County. The final records came from New York in the Northeast region: two records, including one larva, one nymph, and two adults, collected from humans in Sullivan County in the southern part of the state in 1916; and one record including five adults taken from deer in Monroe County in far western New York in 1917.

## 6. Geographic distribution of *I. scapularis* records in the Northeast region (Connecticut, Delaware, Maine, Maryland, Massachusetts, New Hampshire, New Jersey, New York, Pennsylvania, Rhode Island, and Vermont), 1921 to present

The text below presents overviews of the geographic distribution of *I. scapularis* records in the Northeast region from 1921 to 1950 (Section 6.1), 1951 to 1975 (Section 6.2), and 1976 to present (Section 6.3), followed by examples of well documented instances of geographic spread within the Northeast region in recent decades (Sections 6.4–6.7). Generalized directions of spread within the region from early refugia where the tick was known to be present and abundant by 1950 are shown in Fig. 4. Out of the 244 counties in the Northeast region, the cumulative number of counties with collection records of *I. scapularis* (i. e., based on collection locations identifiable to county level) rose sharply from 15 (6% of all counties) by 1950 and 24 (10%) by 1975 to 206 (84%) by 1996 and 243 (>99%) by 2015 (Fig. 5).

### 6.1. Overview of collection records from 1921 to 1950

Records of *I. scapularis* from 1921 to 1950 were geographically very localized in the Northeast. Records from the 1920s were limited to Naushon Island (Dukes County) off the southwest tip of Cape Cod in southeastern Massachusetts and resulted from tick surveillance associated with an effort to control *D. variabilis* by releasing a parasitoid wasp known to parasitize ticks. Bequaert (1945) reported collection of *I. scapularis* adults from white-tailed deer on Naushon Island in 1924 and Larrousse et al. (1928) described collection of *I. scapularis* immatures from white-footed mice (*Peromyscus leucopus*) in 1927. White-tailed deer have reportedly maintained a population ranging from 50 to 500 animals on Naushon Island since the early 1800s (Busby et al., 2008), which helps to explain how a population of *I. scapularis* could persist in this particular location. The tick population documented on Naushon Island in the 1920s also appears to have persisted over time as *I. scapularis* ticks were collected from the same island in 1940 and 1941 (Cobb, 1942; Smith and Cole, 1943; Cooley and Kohls, 1945; USNTC, unpublished data). In a letter described by Cooley and Kohls (1945), C. N. Smith notes regular collection on Naushon Island and the adjacent Nonemesset Island (both part of the Elizabeth Islands) of *I. ricinus scapularis* larvae and nymphs from *Microtus* and *Peromyscus*, adults from deer, and all life stages by drag sampling. *Ixodes scapularis* was also later documented from Naushon Island in 1976–1977 (Piesman et al., 1979; Spielman et al., 1979), 1984–1986 (Piesman et al., 1986; Mather et al., 1987; Telford et al., 1988), and 2018–2020 (Goethert et al., 2021). The well documented Naushon Island scenario illustrates how, against the general backdrop of contraction of the geographic distribution of *I. scapularis* due to decimation of white-tailed deer, local refugia for deer allowed this tick species to persist in specific forested locations which later may have served as starting points (refugia) for recolonization of surrounding areas when deer populations rebounded. The number of such locations in the northern part of the eastern US from which *I. scapularis* could start recolonizing surrounding areas in the latter part of the 1900s is not clear.

Additional Massachusetts collections of *I. scapularis* from 1921 to 1950 (reported by Spielman et al., 1979) were also from or near Cape Cod: East Sandwich in Barnstable County on Cape Cod in 1936 (14 females and three males from fox); Brewster, Chatham, Falmouth, and Hatchville in Barnstable County on Cape Cod in 1948–1949 (11 females and one male from dog and fox, and two females and one male from an unknown source); and Onset and Wareham in Plymouth County just west of Cape Cod in 1949 (three females from dog). The USNTC also includes numerous records of *I. scapularis* (totals of more than 100 nymphs and 200 adults) taken from various hosts in Barnstable and Dukes counties from 1934 to 1946, in some cases likely the same specimens as reported in the published literature. The lone geographic outlier from Massachusetts was one female *I. scapularis* collected from an unknown source in Walpole, Norfolk County, roughly 80 km northwest of Cape Cod in 1949 (Spielman et al., 1979).

The majority of other collection records for *I. scapularis* from the Northeast during 1921–1950 were from another refugium site for white tailed deer (Severinghaus and Brown, 1956): Long Island, Suffolk County in New York. A series of collection records from different locations on Long Island (including Greenport, Montauk Point, Sag Harbor, and Southampton) from 1945 to 1950 included *I. scapularis* nymphs taken from various wild mammals as well as adults taken by drag sampling or from human, rabbit, or skunk (Anastos, 1947; Collins et al., 1949; Spielman et al., 1979; Persing et al., 1990; USNTC, unpublished data). This appears to represent another well documented established population of *I. scapularis* along the Atlantic Coast in the Northeast, together with the previously known population in the Cape Cod area roughly 300 km to the north. There are a few additional records of *I. scapularis* from the Northeast but some of these could be examples of travel-related tick exposures based on the scarcity of collection detail: one female taken from a human in Brooklyn, New York City (Kings County) in 1930; one female taken from a human in Potter County in north-central Pennsylvania in 1938; ticks of unknown numbers, life stages, and sources reported in 1945 from Delaware and Philadelphia counties in southeastern Pennsylvania; and a tick taken from a dog in 1948 in Washington County in coastal Rhode Island (Hyland and Mathewson, 1961; Spielman et al., 1979; Pak et al., 2019). A more intriguing record came from New Haven, New Haven County in Connecticut where a male *I. scapularis* was taken from a white-footed mouse in 1933 (Spielman et al., 1979). New Haven is located directly to the north of Long Island, separated by the Long Island Sound, and thus in proximity to the locations on eastern Long Island where *I. scapularis* was documented in the 1940s. There is also another record from New Haven County in 1938: one female *I. scapularis* taken from a dog (USNTC, unpublished data). One final record of note is an *I. scapularis* nymph taken from an eastern cottontail rabbit in 1933 in Mercer County, New Jersey (USNTC, unpublished data).

### 6.2. Overview of collection records from 1951 to 1975

Records of *I. scapularis* from 1951 to 1975 in the Northeast continue to include Long Island, Suffolk County in New York and the Cape Cod area (Barnstable, Dukes, and Nantucket counties) in Massachusetts, with specimens reported from 1962 to 1975 and representing both immatures and adults taken via drag sampling or collection from various hosts, including humans, dogs, white-footed mice and white-tailed deer (Good, 1973; Spielman, 1976; McEnroe, 1977; Spielman et al., 1979; USNTC, unpublished data). There also are records of *I. scapularis*, including all life stages, from wildlife for four counties in Rhode Island from 1954 to 1957: Bristol County (10 adults from white-tailed deer); Newport County (two larvae and 16 nymphs from rodents, and 41 adults from white-tailed deer); Providence County (12 adults from white-tailed deer); and Washington County (nine adults from opossum, fox, and white-tailed deer) (Hyland and Mathewson, 1961; Spielman et al., 1979; USNTC, unpublished data). Hyland and Mathewson (1961) also noted having seen specimens of *I. scapularis* taken from cats and dogs from the same counties. Additional records from coastal areas of the Northeast include one female from a dog in 1971 with probable exposure on Manursing Island in Westchester County, New York, in the Long Island Sound; and one female from a human taken in 1961 in Clinton, Middlesex County on the Connecticut side of the Long Island Sound (Spielman et al., 1979). Collectively, these records suggest that *I. scapularis* occurred in various locations along the Atlantic Coast from New York to Connecticut, Rhode Island, and southern Massachusetts by the early 1970s. Inland collections of *I. scapularis* were reported from Townsend in Middlesex County, Massachusetts in 1955 (one female from a human); Leverett, Franklin County in Massachusetts in 1956 (ticks of unknown number and life stage taken from raccoons); and Hartford County, Connecticut in 1966 (one female taken from a human) (Parson, 1962; Spielman et al., 1979; USNTC, unpublished data). One final record of note is an *I. scapularis* male from an unknown host and location in New Jersey in 1968 (USNTC, unpublished data). Moreover, Kellogg et al. (1971) reported *I. scapularis* taken from white-tailed deer in Maryland and Massachusetts from 1963 to 1970. Finally, *I. scapularis* was recorded on rare occasions in USDA annual tick surveys from 1963 to 1975 in Connecticut, Maryland, Massachusetts, New York, and Rhode Island (USDA, Animal and Plant Health Inspection Service; unpublished data).

### 6.3. Overview of collection records from 1976 to present

Records of *I. scapularis* from 1976 to present in the Northeast are too plentiful to describe in detail here, and we refer to previous summaries by Dennis et al. (1998) and Eisen et al. (2016a); counties classified as established for *I. scapularis* in these studies are depicted in Fig. 3. There is no doubt that heightened interest in *I. scapularis* during this time period resulted in a dramatic increase in surveillance efforts across the region, reflected in the tenfold increase in the number of counties with records of this tick from 24 by 1975 to 206 by 1996.

The sections below provide examples from the region for how *I. scapularis* was documented to spread from the 1980s to present. Additional indications of spread of *I. scapularis* in the Northeast came from a recent phylogenetic reconstruction of the emergence and spread of a disease agent, Powassan virus, vectored by this tick in the northeastern US (Vogels et al., 2023). The analyses suggest that the virus emerged in *I. scapularis* ticks in southern New York and Connecticut by the 1950s. The virus then spread northward at a rate of approximately 3 km per year, with some long-distance migration events noted, and reached Maine around 1990. These findings mirror the spread of *I. scapularis* documented below and suggest increased spread of the tick and its associated pathogens from 1950 through the 2010s.

### 6.4. Westward and northward expansion across New York State from the Hudson Valley in southeastern New York

By the mid-1980s, *I. scapularis* was common on Long Island in southeastern New York and in Westchester County, located in the southern Hudson River Valley due west of Long Island (Bosler et al., 1984; Falco and Fish, 1988). Established populations were first identified further north in upstate New York (Salem, Washington County) with the detection of ticks on hunter-killed deer in 1985 (Anderson et al., 1987). By 1996, established *I. scapularis* populations were reported from roughly the eastern quarter of the state, with records from each of New York’s easternmost counties bordering Connecticut, Massachusetts, and Vermont; sporadic established populations also were reported in far western New York (Dennis et al., 1998; Fig. 3).

From 1999 to 2009, incidence of Lyme disease increased substantially in the Hudson Valley with expansion of reported cases from south to north (Khatchikian et al., 2015). To track acarological risk of exposure to vector ticks, *I. scapularis* were collected by drag sampling at four sites along a south-north transect along the Hudson Valley in 2004, 2007 and 2009. Phylogenetic analysis of ticks collected during this study suggested that observed detection of the tick in new areas in the northern Hudson River valley was attributable to recent colonization, rather than increases in previously undetected populations. In other words, there was evidence of recent and rapid expansion of the tick through a series of progressive local migration events from the southern to northern Hudson River Valley (Khatchikian et al., 2015). A later study (O’Keeffe et al., 2022) on *I. scapularis* in the Hudson Valley and other parts of New York came to a similar conclusion: ticks were generally most closely related to ticks from the same and nearby locations, although there also was some evidence of spread to more distant locations.

Two other studies with sampling sites distributed more broadly across New York provided additional insights into changing patterns of geographic distribution and local density of *I. scapularis*. From 2004 to 2006, nymphal drag sampling was conducted at 21 forested sites across the state of New York; established, high density populations were documented in the southeast with the distribution spanning further north and west than previously described (Diuk-Wasser et al., 2010). All eight of the 21 sites where *I. scapularis* nymphs were not detected were in far northern and western New York. Between 2008 and 2018, nymphal drag sampling was conducted at 532 sites across the state (Tran et al., 2021). *Ixodes scapularis* ticks were initially detected among these sites in 33% of New York counties in 2008, whereas by 2017 the tick was detected in more than 90% of sampled counties. The early sampling confirmed established *I. scapularis* populations with high nymphal densities in the southeastern portion of the state (Hudson River Valley), and the later sampling indicated spread and proliferation to the remainder of the state over the ten-year observation period. Throughout the study, nymphal densities remained high in the southeastern portion of the state; rates of increases in density were greatest in newly detected areas in northern and western sections of the state.

### 6.5. Expansion into and within Pennsylvania

Published records of *I. scapularis* are lacking from Pennsylvania from 1945 to 1987 (Pak et al., 2019), despite some notable sampling efforts over this time period. During the summers of 1963–1968, ticks were collected by dragging, flagging, small mammal collections, retrospective review of museum records, and passive surveillance involving veterinarians, pest control operators, and “housewives and other citizens” (Snetsinger, 1968). Among approximately 1200 submissions, 20 tick species were identified from 51 of Pennsylvania’s 67 counties, but *I. scapularis* was not detected in the state. Two decades later, in 1989, an effort was made to define areas of elevated risk for Lyme disease in the northeastern US via inspection of hunter-killed deer for ticks and canine surveillance for *Bo. burgdorferi* s.s. antibodies (Daniels et al., 1993). Although sampling was limited in Pennsylvania, *I. scapularis* was not detected on deer and *Bo. burgdorferi* s.s. antibodies were not detected in dogs collected in this state. Based on deer infestation and canine serology, the authors defined a zone of Lyme disease endemicity from central Massachusetts south to central Maryland with risk declining westward and northward (Daniels et al., 1993). However, in a retrospective paper on Pennsylvania ticks, Pak et al. (2019) presented collection records from 1987 to 1989 where single *I. scapularis* were taken from a number of humans or dogs in counties located in far southeastern Pennsylvania (Berks, Chester, Delaware, and Montgomery) and far northwestern Pennsylvania (Elk, Erie, and Warren).

These disparate locations for collection of *I. scapularis* are intriguing as they are separated by the Appalachian Mountains. Far-eastern Pennsylvania borders on New Jersey, where *I. scapularis* was collected already in 1981 from deer in multiple counties in the western part of the state (Gloucester, Hunterdon, Mercer, and Salem) bordering on eastern Pennsylvania, albeit separated by the Delaware River (Schulze et al., 1984a, 1984b). There is no similar readily explained route for invasion of *I. scapularis* in the 1980s to far northwestern Pennsylvania, separated from the eastern parts of Pennsylvania and New York by the Appalachian Mountains and bordering to the west and south by areas of Ohio and West Virginia without known populations of *I. scapularis* in the 1980s. Intriguingly, far northwestern Pennsylvania is close to Long Point in Ontario, Canada where a local population of *I. scapularis* was documented already in 1972–1973 (Watson and Anderson, 1976), and Dennis et al. (1998) reported established populations of *I. scapularis* from two counties (Cattaraugus and Erie) in far western New York located between Long Point and the tick focus in northwestern Pennsylvania. Other possible explanations for the emergence of *I. scapularis* in northwestern Pennsylvania include deer and *I. scapularis* having persisted over time in local refugia or the tick being introduced via restocking of infested deer sourced from areas where *I. scapularis* was present. Restocking of deer in Pennsylvania included bringing in deer from other states (Kentucky, Maine, Michigan, New Hampshire, New Jersey, North Carolina, and Ohio) from 1906 to 1924, and within-state translocations of deer from 1914 to 1967 (McDonald and Miller, 2004).

Collection records from Pennsylvania then accumulated rapidly from 1990 to 1995, with *I. scapularis* taken primarily from humans and dogs (Pak et al., 2019). By 1996, *I. scapularis* was reported to be present in 49 of 67 counties in Pennsylvania, and to meet criteria for establishment (i. e., at least six individuals of a single life stage or at least two life stages detected in the county) in a subset of counties located in the far-eastern part of the state (Berks, Bucks, Delaware, Lancaster, Monroe, Montgomery, Northampton, Pike, Philadelphia, Wayne, and York) or in the western part of Pennsylvania on the other side of the Appalachian Mountains (Blair, Butler, Clearfield, Elk, Erie, McKean, Mercer) (Dennis et al., 1998). Field surveys between 2000 and 2014, focused in part on detection of human pathogens in ticks, generated additional information about *I. scapularis* in Pennsylvania. Courtney et al. (2003) examined hundreds of *I. scapularis* adults taken from deer in 2000–2001 in Erie County in the northwestern part of the state and Chester and Delaware counties in the southeast. Diuk-Wasser et al. (2010) conducted drag sampling in 17 forested sites across Pennsylvania from 2004 to 2006; *I. scapularis* nymphs were detected in eight sites located primarily in the eastern portion of the state, though the tick and *Bo. burgdorferi* s.s. also were detected in two western counties. Based on records from that study, a climate model was developed that scored western Pennsylvania as low risk for Lyme disease (Diuk-Wasser et al., 2010). However, incidence of Lyme disease doubled in the northwestern portion of the state and more than tripled in the southeast from 2010 to 2013 (Hutchinson et al., 2015). These findings prompted a statewide survey of all 67 counties to assess the prevalence of human pathogens (*A. phagocytophilum, Ba. microti*, and *Bo. burgdorferi* s.s.) in *I. scapularis* adults collected from 2012 to 2014 (Hutchinson et al., 2015). By that time, *I. scapularis* was established in all Pennsylvania counties and each of the pathogens were detected and prevalent across the state, though infection rates were marginally lower in western Pennsylvania.

### 6.6. Northward expansion from Massachusetts into the bordering states of Maine, New Hampshire, and Vermont

The earliest records of *I. scapularis* from Maine (Piscataquis and Somerset counties), New Hampshire (Grafton County), and Vermont (Caledonia County) were adult ticks taken from white-tailed deer in 1985 (Anderson et al., 1987). Soon thereafter, one female *I. scapularis* was collected in 1986 from a human in Lincoln County in coastal Maine (USNTC, unpublished data), and 17 nymphs and one female were collected in 1987 by flagging in Acadia National Park (Hancock County) in coastal Maine, with *Bo. burgdorferi* s.s. detected from two nymphs (Ginsberg and Ewing, 1988). A passive statewide tick surveillance (tick identification) program was then initiated in 1989 (Smith et al., 1992). Submissions of *I. scapularis* increased sharply through the 1990s and early 2000s with submissions originating first from towns situated within 32 km of Maine’s southern and mid-coast. Increasing numbers were submitted from this region over time, with noted increases in submissions from sites further inland and northward, primarily along river corridors (Rand et al., 2007). These spatial and temporal trends in expansion of the tick’s range were mirrored by similar increases in reported cases of Lyme disease (Rand et al., 2007). By 2021, *I. scapularis* was present in each of Maine’s 16 counties; both the numbers of ticks submitted for identification and testing and the prevalence of human pathogens (*A. phagocytophilum, Ba. microti*, and *Bo. burgdorferi* s.s.) was found to decrease along a gradient from southern to northern Maine (Rounsville et al., 2021).

The initial detection of *I. scapularis* in New Hampshire and Vermont in 1985 was followed by a single record of a female collected from a human in coastal Rockingham County in New Hampshire in 1989 (USNTC, unpublished data). There is no further documentation of the tick from these two states until nymphs were collected in low numbers (<20) by drag sampling in single sites in the southern parts of Vermont and New Hampshire from 2004 to 2006 (Diuk-Wasser et al., 2010). No *I. scapularis* were collected from sites in the northern parts of the states in that study. Increasing numbers of Lyme disease cases in New Hampshire motivated a statewide survey of *I. scapularis* adults and *Bo. burgdorferi* s. s. in 2007, with tick collection locations in all 10 counties of the state (Walk et al., 2009). Not surprisingly, ticks were abundant in the southeastern, coastal part of the state where Lyme disease incidence was highest, less abundant or absent from inland sites in the southwest, and no ticks were collected in inland sites to the north. Moreover, infection prevalence with *Bo. burgdorferi* s.s. was highest in the counties with the highest Lyme disease incidence. Recent studies have reported records of *I. scapularis* from all 10 counites in New Hampshire by 2017, with collections being most common in the southeast and least common to the north (Eaton, 2023; Fernández-Ruiz et al., 2023). Regular future tick surveys across New Hampshire are of interest to track the proliferation of *I. scapularis* in the state.

In Vermont, the number of Lyme disease cases reported annually increased from 105 in 2005 to 623 in 2012 and more than 1000 cases were reported in 2017 with a strong geographical trend of higher incidence in the southwestern corner of the state to near zero in the northeastern section of the state (Serra et al., 2013; Allen et al., 2019), suggesting recent colonization of the tick vector from the south. To track trends in the density of host-seeking *I. scapularis* in Vermont, drag sampling was conducted biweekly from June to December 2011 and from March to June 2012 at five forested sites along a north-south gradient that followed the Connecticut river, which runs along the eastern border of Vermont (Serra et al., 2013). All life stages of *I. scapularis* were collected from the three southernmost sites, while only a single nymph was collected at the next most northern site and no *I. scapularis* of any life stage were collected from the most northern site. Another recent study conducted from 2016 to 2018 in the Champlain Valley of west-central Vermont documented abundant populations of *I. scapularis* (Allen et al., 2019). Regular future tick surveys across Vermont are of interest to track the proliferation of *I. scapularis* in the state.

### 6.7. Records from the tri-state area of New Jersey, Delaware, and Maryland

As noted previously, there was very limited information on *I. scapularis* from the tri-state area of New Jersey, Delaware, and Maryland in the southernmost part of the Northeast region up to 1975. In New Jersey, *I. scapularis* was documented from deer in Cape May County on the coast in 1976 (Spielman et al., 1979). The tick was subsequently collected from a human, deer, and various other mammals in Monmouth County and elsewhere in the state from 1978 to 1982 (Schulze et al., 1984a, 1984b, 1985, 1986). This included an effort in 1981 to document *I. scapularis* in New Jersey by examination of hunter-killed deer, which revealed that the tick was already widespread in the state (Schulze et al., 1984a, 1984b). In Delaware, a record of an adult *I. scapularis* taken from a dog in New Castle County in 1982 (USNTC, unpublished data) was followed by documentation of the tick from hunter-killed deer in all three counties of the state in 1988, including collection of more than 2800 adult *I. scapularis* (Wolfe et al., 1994). In Maryland, all life stages of *I. scapularis* were documented from Assateague Island in 1980–1981 (Coan and Stiller, 1986), and there were two records of small numbers of *I. scapularis* taken from horse and white-tailed deer in unknown locations in the state from 1982 to 1983 (USNTC, unpublished data). Additional records accumulated from multiple counties (Anne Arundel, Calvert, Frederick, Howard, Montgomery, Prince Georges, Talbot, and Worcester) in Maryland from 1986 to 1988, most often with small numbers of adult *I. scapularis* collected from humans, cats, and deer (USNTC, unpublished data). A statewide effort to examine hunter-killed deer in Maryland in 1989 then yielded more than 3000 *I. scapularis* adults from counties across the state, with the highest tick loads on deer from the coastal portion of the state (Amerasinghe et al., 1992). It is not clear whether *I. scapularis* persisted in one or several local refugia in the New Jersey-Delaware-Maryland area (remnant populations of white-tailed deer persisted in far western Maryland and perhaps also in parts of New Jersey; McDonald and Miller, 2004) or was introduced from elsewhere via natural host movement or translocated deer. As the *I. scapularis* in the New Jersey-Delaware-Maryland area are northern population ticks, it seems likely they may have invaded southward from New York.

## 7. Geographic distribution of *I. scapularis* records in the Upper Midwest region (Iowa, Michigan, Minnesota, and Wisconsin), 1921 to present

The text below presents overviews of the geographic distribution of *I. scapularis* records in the Upper Midwest region from 1921 to 1950 (Section 7.1), 1951 to 1975 (Section 7.2), and 1976 to present (Section 7.3), followed by examples of well documented instances of geographic spread within or into the Upper Midwest region in recent decades (Sections 7.4–7.8). Generalized directions of spread within the region from a refugium where the tick was known to be present and abundant by 1970 are shown in Fig. 4. Out of the 341 counties in the Upper Midwest region, the cumulative number of counties with collection records of *I. scapularis* rose from 1 (<1% of all counties) by 1950 and 10 (3%) by 1975 to 117 (34%) by 1996, 193 (57%) by 2015, and 284 (83%) by 2022 (Fig. 5).

### 7.1. Overview of collection records from 1921 to 1950

The only record of *I. scapularis* from 1921 to 1950 in the Upper Midwest region came from Clayton County in Iowa in 1939, where two females were collected from an unknown source (Spielman et al., 1979).

### 7.2. Overview of collection records from 1951 to 1975

Records of *I. scapularis* from 1951 to 1975 in the Upper Midwest started to accumulate when a female tick was recorded from a human in Lincoln County in the north-central part of Wisconsin in 1965 (Jackson and Defoliart, 1970). Subsequent work from 1967 to 1968 documented *I. scapularis* collected by drag sampling (13 adults) and from human and dog (three adults) in Chippewa County. and from deer (97 adults) in Barron, Burnett, Rusk, Sawyer, and Taylor counties, all in northwestern Wisconsin (Jackson and Defoliart, 1970; Spielman et al., 1979; USNTC, unpublished data). In 1970, local residents reported the tick to be a pest in northwestern Wisconsin for only the past five to six years, suggesting either recent invasion of the area or increases in densities of existing local tick populations. It also should be noted that the white-tailed deer had persisted in the northern part of Wisconsin during the late 1800s and early 1900s while they became scarce to absent from the southern part of the state by 1910 (Dahlberg and Guettinger, 1956), suggesting there may have been local refugia sites for *I. scapularis* in or near the areas where the tick was becoming more abundant in northwestern Wisconsin in the late 1960s. Focal tick populations were described in “potholes” or depressions that resulted from glacial outwash, which are commonly associated with lowland forests in Wisconsin, with expansion of lower abundance populations to the east (Jackson and DeFoliart, 1970). Additional collections of *I. scapularis* included one female from a dog in 1971 from either Racine or Kenosha counties in southeastern Wisconsin (Amin, 1973); ticks of unknown life stage and number from black bears in 1975 from Sawyer County in northwestern Wisconsin (Manville, 1978); one male from a dog in 1975 in Pine County in easternmost Minnesota, located adjacent to Burnett County in northwestern Wisconsin (Spielman et al., 1979); and two females from dog(s) in 1975 from Barron County in northwestern Wisconsin (USNTC, unpublished data). These records collectively indicate a focus of *I. scapularis* in northwestern Wisconsin as early as the 1960s, potentially extending into easternmost Minnesota. Finally, *I. scapularis* was recorded on rare occasions in USDA annual tick surveys from 1963 to 1975 in Iowa, Minnesota, and Wisconsin (USDA, Animal and Plant Health Inspection Service; unpublished data).

### 7.3. Overview of collection records from 1976 to present

In Wisconsin, reports of sporadic cases of erythema chronicum migrans in the late 1960s (Scrimenti, 1970) and Lyme arthritis in the late 1970s (Dryer et al., 1979) were followed by the description of a large number of Lyme disease cases from 1980 to 1982 (Davis et al., 1984). Together with data from the Northeast implicating *I. scapularis* as a vector of *Bo. burgdorferi* s.s., these Lyme disease cases motivated efforts to better define the geographic distribution of *I. scapularis* across the Upper Midwest from the early 1980s onward. Based on the findings of these efforts, the tick appears to have spread from the initially detected focus in northwestern Wisconsin in all geographic directions, with rates of spread influenced by geographic barriers. Areas with fragmented forests or large water masses impeded, but did not prevent, colonization by *I. scapularis* of forested patches throughout the upper Midwest over a half century (circa 1970–2020). The dramatic increase in surveillance efforts across the region during the 1980s and 1990s (see references in Sections 7.4–7.7) is reflected in a tenfold increase in the number of counties with records of this tick from 10 by 1975 to 117 by 1996. However, as outlined in the sections below, there are clear examples of true spread and proliferation based on surveillance that was initiated while the tick was still absent or present only in very low numbers. By 2022, *I. scapularis* was widely distributed across the forested portions of the Upper Midwest (CDC, 2023b; Fig. 3)

### 7.4. Expansion within Wisconsin

A series of studies reported expanding distribution of *I. scapularis* in Wisconsin based on state-wide screening of hunter-killed deer from 1979 to 1982 (Davis et al., 1984), in 1981 and 1989 (French et al., 1992), in 1994 (Riehle and Paskewitz, 1996), and from 2008 to 2009 (Lee et al., 2013). Initial deer surveys from 1979 to 1982 found *I. scapularis* to be widespread in the northwestern, southwestern, and west-central parts of the state but very scarce in the east, where it was collected only from a few deer in the southeast (Davis et al., 1984; French et al., 1992). The highest numbers of ticks infesting deer were reported from northwestern Wisconsin. Concordant with these findings, Lyme disease case reporting from 1980 to 1982 revealed that exposures to vector ticks most commonly occurred in northwestern counties (Davis et al., 1984). Small and medium-sized mammal trapping also was conducted during 1982–1983 in four regions of Wisconsin to assess the distribution of ticks and Lyme disease spirochetes: northwestern Wisconsin where Lyme disease was endemic (Spooner and Washburn counties), west-central Wisconsin where Lyme disease cases were recently diagnosed (Monroe County), southwestern Wisconsin where the tick was present, but Lyme disease was not documented (Iowa and Sauk counties), and east-central Wisconsin where neither ticks or Lyme disease cases were reported (Fond du Lac and Sheboygan counties) (Godsey et al., 1987). *Ixodes scapularis* were collected from trapped mammalian hosts in each region except the east-central region.

Selected sites where deer had been sampled in 1981 (French et al., 1992) were then resampled in 1989 (French et al., 1992) and 1994 (Riehle and Paskewitz, 1996). This included reference sites in western and central Wisconsin but focused primarily on sites to the east where the tick was absent or scarce in 1981. Deer sampling in 1989 and 1994 revealed southward expansion of *I. scapularis* in western and central Wisconsin, as well as an increasing proportion of deer infested by this tick in the western part of the state compared to 1981. *Ixodes scapularis* was still absent from deer in most resampled sites in the eastern third of the state in 1989 and 1994, although a few deer were infested with small numbers of ticks in one site in the southeast and one far eastern site (French et al., 1992). The 1994 survey also included additional sites in central Wisconsin, revealing an increase in *I. scapularis* infestations on deer in the central portion of the state (the Wisconsin River Valley) that had previously represented the eastern edge of the tick’s recorded range in Wisconsin, as well as documenting records in counties further south and east than seen previously (Riehle and Paskewitz, 1996). Extensive rodent trapping and flag sampling was conducted in southeastern Wisconsin and northeastern Illinois from May through August 1989; no *I. scapularis* were detected, increasing confidence in the assumption that the tick was not yet established in these locations at that time (Callister et al., 1991). During a similar time period (1988 and 1989), mice and deer were inspected for *I. scapularis* along a west to east transect in south-central Wisconsin. An abrupt west to east decline in tick abundance was documented (French, 1995).

However, Walker et al. (1996) described an established, localized population in Marinette County in far northeastern Wisconsin, outside of the previously described range, based on flag sampling and rodent trapping in 1993. This county is located along the western shore of Lake Michigan and borders on Menominee County in Michigan, where a focus of *I. scapularis* previously had been documented from 1988 to 1992 (Strand et al., 1992; Walker et al., 1994). It is possible that *I. scapularis* had persisted in a refugium along this part of Lake Michigan, separate from the refugium in northwestern Wisconsin.

More recent surveys of hunter-killed deer conducted in 2008 and 2009 (Lee et al., 2013) revealed widespread occurrence of *I. scapularis* throughout eastern Wisconsin, though the distribution was more patchy, and abundance often lower in eastern counties compared to other parts of the state. This may reflect more recent invasion, or poorer quality habitat compared with western Wisconsin (Lee et al., 2013). By 2015, *I. scapularis* had been recorded from nearly all Wisconsin counties (Eisen et al., 2016a).

### 7.5. Westward expansion from Wisconsin to Minnesota

A limited number of tick surveys have documented westward expansion of *I. scapularis* from its focus in northwestern Wisconsin into neighboring Minnesota, or alternatively documented that the original focus could have originated in Minnesota. To assess the distribution of *I. scapularis* in Minnesota, in September and October of 1985 and 1986 ticks were collected by ruffed grouse hunters using dogs (Drew et al., 1988)*. Ixodes scapularis* ticks were found most commonly in east-central Minnesota (neighboring the previously described focal population in northwestern Wisconsin) and occasionally detected in the southeastern portion of the state. None were detected at surveillance sites in central, western, or northern sites. Their absence in the large number of sites surveilled in the north builds confidence that the tick was absent or rare in that portion of Minnesota in the mid-1980s. A survey of ticks on migratory birds in the Saint Croix River Valley in 1987 documented a well-established population of *I. scapularis* in Pine County, Minnesota, bordering on Burnett County in northwestern Wisconsin where *I. scapularis* was recorded in the late 1960s (Jackson and Defoliart, 1970; Weisbrod and Johnson, 1989). These data suggest the presence of the tick in eastern Minnesota well before the survey in the mid-1980s, but the tick’s absence in the north, central and western sites suggests that by the mid-1980s the western edge of the distribution was limited to eastern Minnesota.

As of 1996, only nine Minnesota counties (Anoka, Carver, Chisago, Dakota, Morrison, Pine, Ramsey, Scott, and Washington) were classified as having established populations of *I. scapularis* (Dennis et al., 1998; Fig. 3). These counties were located primarily in the Minneapolis-Saint Paul region and slightly north along the Wisconsin border, and northwest in central Minnesota. However, although not meeting the criteria for establishment, *I. scapularis* was reported from another 21 counties that were widely distributed in the northeastern, north-central, northern and southeastern portions of the state. From 2004 to 2006, Diuk-Wasser et al. (2010) conducted drag sampling targeting host-seeking *I. scapularis* nymphs in 26 Minnesota sites, with nymphs collected primarily in the central third of the state extending northwest from the focal population described along the border with Wisconsin. In 2015, with the aim of describing geographical trends in the distribution and abundance of host-seeking *I. scapularis* nymphs, 81 forested sites in Minnesota were drag sampled (Johnson et al., 2018). Sites were distributed across the state, but sites in the southwest and far western portions of the state were few, owing to relatively low forest coverage in those regions. The highest densities of host-seeking nymphs were detected in the Minneapolis-Saint Paul region, with the distribution of other high-density sites trending northwestward, following a trend of higher densities in presumably longer-established sites. Consistent with these acarological trends, from 1999 through 2011 a steady increase in Lyme disease and anaplasmosis cases were reported along a northwesterly trajectory, with notable increases, but lower incidence, also reported in the southeast (Robinson et al., 2014). Because we do not expect substantial changes in human behavior that would modify risk of exposure to ticks or anomalies in case reporting that would follow a northwest trend in increasing incidence over time, the increasing trend in tick-borne infections is likely consistent with increasing densities of infected ticks in these regions of the state.

By 2015, 45 of Minnesota’s 87 counties were classified as having established populations of *I. scapularis*; these were located along the entirety of the state’s eastern border with Wisconsin spanning northwest across most of the state (Eisen et al., 2016a; Johnson et al., 2016). A total of 42 counties lacked establishment records of the tick; these were located along the western and southwestern borders of the state where agricultural land predominates and forests are relatively sparse (Fig. 3).

### 7.6. Westward expansion from Wisconsin or Illinois to Iowa

An initial effort was made from 1989 to 1991 to survey *I. scapularis* across Iowa based on a combination of drag sampling, small mammal trapping, checking of hunter-killed deer, and submissions from the public and veterinary and medical practitioners (Novak et al., 1991; Bartholomew et al., 1992). The tick was found to already be widely distributed at low abundance throughout the eastern part of the state, which is separated on the eastern border from neighboring southwestern Wisconsin and northwestern Illinois by the Mississippi River. Incursions of *I. scapularis* across the Mississippi River into Iowa may have occurred from areas in southwestern Wisconsin, where the tick was collected from small mammals already in 1982–1983 (Godsey et al., 1987), and from far northwestern Illinois, where *I. scapularis* was recorded by drag sampling and from hunter-killed deer in 1987–1988 (Bouseman and Kirkpatrick, 1988; Bouseman et al., 1990).

Statewide passive surveillance efforts then documented the distribution of *I. scapularis* in Iowa from 1990 to 2013 (Lingren et al., 2005; Oliver et al., 2017). From 1990 to 1996, *I. scapularis* submissions were derived from counties along the Mississippi River on the eastern border of the state; a marked westward expansion into central Iowa was noted from 1997 to 2002 (Lingren et al., 2005) and continued from 2004 to 2013 (Oliver et al., 2017). Iowa’s landcover is transitional between hardwood forest, prairie remnants and extensive agriculture; only 7% of the land remains forested, limiting the amount of suitable habitat for this woodland-associated tick. While deer are abundant throughout the state, their abundance is greatest along waterways, suggesting continued spread of *I. scapularis* primarily along riparian corridors.

### 7.7. Michigan’s Upper Peninsula

The USNTC includes two early records of *I. scapularis* from Michigan: two adults collected from dog(s) in unknown locations in the state in 1982, and two adults collected from white-tailed deer in Menominee County in the Upper Peninsula in 1985 (USNTC, unpublished data). *Ixodes scapularis* was then collected, in low numbers (10 adults), from various areas of the Upper Peninsula of Michigan in 1986 (Strand et al., 1992), and one adult was recovered from a human in 1988 in Delta County, which borders on Menominee County (USNTC, unpublished data). During 1988–1992, a focus of *I. scapularis* was documented in Menominee County, where hundreds of immature ticks were collected from rodents and more than one thousand adults were collected by flag sampling (Strand et al., 1992; Walker et al., 1994). As Michigan’s Upper Peninsula, including Menominee County itself, shares a land border with Wisconsin, the tick may have reached this part of Michigan via expansion from eastern Wisconsin. However, as *I. scapularis* was not common in eastern Wisconsin by the late 1980s, it cannot be ruled out that the Menominee County focus represent a refugium site for the tick or that it had been introduced from elsewhere via restocking of deer.

From 1985 to 1996, a passive tick surveillance program collected ticks submitted by citizens in Michigan. The number of submitted *I. scapularis* increased over time, from less than 10 per year during 1985–1988 to 27–78 per year during 1989–1996. After removing submissions associated with out-of-state travel, 87% of the *I. scapularis* ticks submitted were recovered from four counties in the western part of the Upper Peninsula (Delta, Dickinson, Gogebic, and Menominee) and the vast majority of those ticks were from Menominee County. *Ixodes scapularis* was only occasionally detected in submissions from the eastern part of the Upper Peninsula. Based on drag sampling conducted from 2004 to 2006, host-seeking nymphs were recovered only from one site in the western part of the Upper Peninsula; none were collected from four sites situated in the central and eastern portions of the Upper Peninsula (Diuk-Wasser et al., 2010). By 2022, nearly all counites in the Upper Peninsula were categorized as established for *I. scapularis* (CDC, 2023b; Fig. 3).

### 7.8. Northward expansion from Indiana to Michigan’s Lower Peninsula

The most populated counties in Michigan are located in the Lower Peninsula, where submissions of *I. scapularis* by residents from 1985 to 1996 were infrequent, but submissions of other tick species were common (Walker et al., 1998). The authors concluded there was no evidence to suggest the tick was established in the Lower Peninsula as late as 1996. An established population of *I. scapularis* was first detected in Michigan’s Lower Peninsula in 2001–2002 from the far southwest of the state (the adjoining Berrien, Van Buren, and Allegan counties along Lake Michigan), based on collections of ticks from hunter-killed deer and small mammals as well as drag sampling (Foster, 2004). Drag sampling from 2002 to 2003 in additional counties directly to the east or north produced no *I. scapularis*. The location of the *I. scapularis* population in the extreme southwest of Michigan’s Lower Peninsula is suggestive of northward spread from neighboring Indiana in the Ohio Valley where *I. scapularis* already was established in the far north of the state by 1990 (Pinger et al., 1991). Previous range expansion of *I. scapularis* from southern Wisconsin via Illinois to Indiana, beginning in the 1980s, is described in Sections 9.4 to 9.5.

From 2004 to 2008, host-seeking and small mammal- or bird-infesting ticks were collected along a south to north transect consisting of four sites on the coast of Lake Michigan (Hamer et al., 2010), with the southernmost site in Van Buren County where *I. scapularis* previously had been recorded by Foster (2004). Four inland sites in southwestern and south-central Michigan also were sampled. Over the five-year study period, northward spread of the tick was documented for the sites along the coast of Lake Michigan based on small animal captures and drag sampling. Prevalence and intensity of small mammal infestations, as well as density of host-seeking nymphs, generally decreased from south to north, but there were clear increases in tick abundance for the northern sites over the study period. Sampling of the inland sites produced only a few (*n* = 7) *I. scapularis* immatures from more than 700 examined mammals and more than 500 birds; and only a single *I. scapularis* larva was collected by drag sampling (Hamer et al., 2010). A similar general trend for Michigan’s Lower Peninsula was reported by Diuk-Wasser et al. (2010): drag sampling from 2004 to 2006 at 16 sites fairly evenly distributed across the Lower Peninsula produced *I. scapularis* nymphs only from two sites in the far southwest along Lake Michigan.

From 2008 through 2022, established populations of *I. scapularis* were documented across the Lower Peninsula, and along the coasts of Lake Michigan to the west, Lake Huron to the northeast, and Lake Erie in the southeast (CDC, 2023b; Fig. 3). We were unable to identify extensive sampling efforts in eastern Michigan after 2008 that would document emergence of the tick in that region. Therefore, it is not clear if the eastern populations of *I. scapularis* in the state originated from western Michigan, or from Ohio to the south or Ontario, Canada, to the east.

Tracking changes in the expansion of Lyme disease cases in Michigan from 2000 to 2014 mirrors trends of *I. scapularis* range expansion (Lantos et al., 2017). Specifically, over this 15-year period, Lyme disease cases increased more than five-fold in both the Upper Peninsula and Lower Peninsula of Michigan. From 2000 to 2004 when the tick had been recorded as established only in the Upper Peninsula, no significant portion of the 108 Lyme disease cases reported statewide were seen in the Lower Peninsula and cases were concentrated in southern Menominee County. Over the subsequent decade, Lyme disease cases accumulated in the southwestern corner of the Lower Peninsula where the tick first established, then increased northward along the coast of Lake Michigan. Sporadic cases were reported from southeastern lower Michigan from 2005 to 2014, before the tick was documented in far-eastern lower Michigan, but it is worth noting that cases are attributed to county of residence rather than exposure, and some of the highest density human populations are situated in southeastern lower Michigan.

## 8. Geographic distribution of *I. scapularis* records in the Northern Rockies and Plains region states of Nebraska, South Dakota, and North Dakota, 1921 to present

The northwestern edge of the range of *I. scapularis* in the US is located in Nebraska, South Dakota, and North Dakota in the eastern part of the Northern Rockies and Plains region (CDC, 2023b). Wooded riparian corridors present suitable habitat and corridors for spread of the tick in the open plains landscape (Nielsen et al., 2020). The first record of *I. scapularis* from the region came from far eastern South Dakota in 1969 when an unspecified number of ticks were taken from deer in Brookings County, which borders on southwestern Minnesota (McDaniel and Hildreth, 1992). The next records did not come until 1990 from Nebraska, when a single female was collected from a white-tailed deer in Cass County and another female was recovered from a dog with probable local acquisition in Pawnee County (Cortinas and Spomer, 2014). Both counties are located in far southeastern Nebraska, on or near the borders with southern Iowa and northern Missouri. The USNTC includes records of *I. scapularis* from 1991 in South Dakota (one female taken from a dog in Codington County in the far eastern part of the state) and North Dakota (one adult taken from a human in Grand Forks County in the far eastern part of the state) (USNTC, unpublished data). Dennis et al. (1998) then noted presence of *I. scapularis*, below the establishment criteria, in 2 counties (Brookings and Codington) in South Dakota by 1996; both counties are located on or near the border with Minnesota. The next suite of records came from 2005 to 2006 and included single *I. scapularis* ticks collected from dogs with probable local acquisition in Butler County and Lancaster County in far eastern Nebraska (Cortinas and Spomer, 2014). During the same time period, Diuk-Wasser et al. (2010) conducted drag sampling in five forested sites in eastern North Dakota and seven forested sites in eastern South Dakota, but *I. scapularis* was not detected.

More recent efforts have documented *I. scapularis* in all three states. In eastern North Dakota, a survey conducted in 2010 documented established *I. scapularis* populations from four counties (Eddy, Grand Forks, Pembina, and Ramsay) and a small number of ticks also from Rolette and Steele counties (Russart et al., 2014). Moreover, *Bo. burgdorferi* s.s. was detected from one nymph and two adults collected in Grand Forks County (Russart et al., 2014). In far eastern South Dakota, surveillance efforts in 2015–2019 documented *I. scapularis* in three counties to the north (Day, Marshall and Roberts) and three counties along the Missouri River in the south (Clay, Lincoln, and Union) (Maestas et al., 2016, 2018; Black et al., 2021). Two of these counties (Clay and Day) reached the criteria for an established *I. scapularis* population, and *Bo. burgdorferi* s.s. was detected in a total of three adult *I. scapularis* from the two counties (Maestas et al., 2018; Black et al., 2021). In far eastern Nebraska, established populations of *I. scapularis* were documented along the Platte River in Douglas, Sarpy, and Saunders counties in 2019 (Nielsen et al., 2020), and in Thurston County in 2021 (Hamik et al., 2023). Moreover, *Bo. burgdorferi* s.s. was detected from seven *I. scapularis* from Thurston County (Hamik et al., 2023).

Out of the 212 counties in Nebraska, South Dakota, and North Dakota, the cumulative number of counties with collection records of *I. scapularis* was 1 (<1% of all counties) by 1975 and rose to 2 (1%) by 1996, 13 (6%) by 2015, and 21 (10%) by 2022 (Fig. 5). Based on *I. scapularis* being collected primarily from counties in the far eastern parts of all three states, it is reasonable to assume that *I. scapularis* invaded in recent decades from the eastern neighboring states of Minnesota and Iowa.

## 9. Geographic distribution of *I. scapularis* records in the Ohio Valley region (Illinois, Indiana, Kentucky, Missouri, Ohio, Tennessee, and West Virginia), 1921 to present

The text below presents overviews of the geographic distribution of *I. scapularis* records in the Ohio Valley region from 1921 to 1950 (Section 9.1), 1951 to 1975 (Section 9.2), and 1976 to present (Section 9.3), followed by examples of well documented instances of geographic spread into the Ohio Valley region in recent decades (Sections 9.4–9.8; see also Fig. 4). Out of the 667 counties in the Ohio Valley region, the cumulative number of counties with collection records of *I. scapularis* rose from 5 (<1% of all counties) by 1950 and 6 (<1%) by 1975 to 131 (20%) by 1996, 329 (49%) by 2015, and 448 (67%) by 2022 (Fig. 5).

### 9.1. Overview of collection records from 1921 to 1950

Records of *I. scapularis* from 1921 to 1950 in the Ohio Valley region include collections from: (i) Jackson County in far southern Illinois in 1939: four nymphs from lizard (Spielman et al., 1979); (ii) an unspecified location in Indiana in 1939: one male from an unknown source; (Spielman et al., 1979); (iii) Stone and Taney counties in far southern Missouri in 1941–1942: three females and two males from horse(s) and one female from a human (Cooley and Kohls, 1945); and (iv) Montgomery, Stone, and Taney counties in Missouri in 1946–1947: more than 1000 adults and immatures from various hosts, including dogs, cattle, horses, fox, and deer (USNTC, unpublished data). Additionally, Bequaert (1945) reported infestation by *I. scapularis* of a dog in the vicinity of Cleveland in Ohio but did not provide a year of collection. Travel histories were not described for infested humans or domestic animals during this time period. It is, however, not surprising to see *I. scapularis* records from the Ohio Valley region as it is located between the Southeast/South regions where records were plentiful, and the Upper Midwest region where records were very scarce during the same time period.

### 9.2. Overview of collection records from 1951 to 1975

There is one record from 1957 of collection of 28 *I. scapularis* adults from white-tailed deer in St. Louis, Missouri (USNTC, unpublished data). Additional records of *I. scapularis* from 1951 to 1975 in the Ohio Valley region are restricted to occasional collections of specimens in the USDA annual tick surveys from 1963 to 1975, including from Illinois, Kentucky, Missouri, Ohio, and Tennessee (USDA, Animal and Plant Health Inspection Service; unpublished data).

### 9.3. Overview of collection records from 1976 to present

The emergence of Lyme disease in the Northeast and Upper Midwest in the 1980s motivated adjoining states in the Ohio Valley region to start surveillance for *I. scapularis*, for example via examination of hunter-killed white-tailed deer. Such efforts in Illinois, Indiana, and Ohio are described below in Sections 9.4 to 9.6. The states further south in the Ohio Valley region (Missouri, Kentucky, Tennessee, and West Virginia) tended to not initiate such surveillance efforts until later when it was clear that northern populations of *I. scapularis*, and Lyme disease cases, were spreading southward from Illinois, Indiana, and Ohio, or from Pennsylvania in the Northeast. Dennis et al. (1998) listed only a limited number of counties with established populations of *I. scapularis* (see Fig. 3) in the Ohio Valley region by 1996, including 21 counties in southern Missouri, smaller numbers of counties in the northern parts of Illinois (*n* = 4) and Indiana (*n* = 8), two counties (Berkeley and Jefferson) in West Virginia, and a single county (Shelby) in southern Tennessee (for records from Missouri and Tennessee, see also Kollars, 1996; Kollars et al., 1997, 1999).

From 2004 to 2006, Diuk-Wasser et al. (2010) conducted drag sampling for *I. scapularis* nymphs in all the states in the Ohio Valley region. This produced the largest numbers of host-seeking nymphs from Illinois and Indiana, smaller numbers from southern Missouri, and no nymphs from sites in Tennessee, Kentucky, Ohio, or West Virginia. However, it should be noted that the focus on the nymphal activity period (May to September) for the drag sampling effort may have overlooked southern populations of *I. scapularis* as the nymphs tend to quest cryptically and therefore are less likely to be collected by drag sampling compared to nymphs of northern populations, and the seasonal peak activity period for the more readily collected adults of southern populations does not coincide with the nymphal peak. Later studies on *I. scapularis* in the Ohio Valley region are outlined in Sections 9.4 to 9.8. By 2022, *I. scapularis* had been documented to be widely distributed across the Ohio Valley region, albeit a smaller proportion of individual counties had records of the tick compared to the Upper Midwest and Northeast regions (CDC, 2023b; Fig. 5).

### 9.4. Southward expansion from Wisconsin to Illinois

Illinois’ land cover is predominantly agricultural with forest more common in southern reaches of the state and along riparian corridors in the north. Two *I. scapularis* females were collected from two white-tailed deer in Jo Daviess County in far northwestern Illinois in 1987 (Bouseman and Kirkpatrick, 1988). This was followed up in 1988 by inspections of hunter-killed deer across Illinois and drag sampling for *I. scapularis* in five counties (Carroll, Jo Daviess, Ogle, Stephenson, and Winnebago) in the far northwest, bordering on Wisconsin (Bousemann et al., 1990; Nelson et al., 1991). Host-seeking *I. scapularis* were collected only from Ogle County, whereas the tick was collected from deer in Carroll, Jo Daviess, Ogle, and Lee counties in the far northwest, Knox, Mercer, and Rock Island counties in the far west central part of the state, and also in Edgar, Kankakee, and Piatt counties in east central Illinois. The greatest numbers of infested deer (>5 per county) came from counties along the Rock River, which extends from southern Wisconsin westward through northwestern Illinois to the Mississippi River on the border with Iowa. The authors speculated that *I. scapularis* may have invaded Illinois via movement of deer southward from Wisconsin along the Rock River, or along the Mississippi River which marks Iowa’s border with Wisconsin to the north and Illinois to the south. Both rivers flow through areas in southern Wisconsin where *I. scapularis* was recorded from deer in the 1980s (French et al., 1992). Another study conducted during the same time period (1988–1990) near Chicago in northeastern Illinois failed to collect *I. scapularis* from rodents or by flag sampling (Callister et al., 1991). Dennis et al. (1998) listed only four counties (Carroll, Monroe, Ogle, and Rock Island) in Illinois as having established populations of *I. scapularis* by 1996 (Fig. 3).

Additional surveys of hunter-killed deer from 1998 to 2003 then demonstrated infestation with *I. scapularis* in numerous counties along the Rock River and along the Illinois River, which extends roughly diagonally from northeastern to southwestern Illinois, from the mid-1980s through 2000 (Cortinas et al., 2002; Cortinas and Kitron, 2006). From 2004 to 2006, Diuk-Wasser et al. (2010) recorded *I. scapularis* nymphs by drag sampling from sites in northern and east-central Illinois but not from the southwestern part of the state. Most recently, an extensive review of first county records of *I. scapularis* in Illinois indicate spread from north to south with initial detection of the tick from 1987 to 1992 restricted to the northern part of the state but with records from numerous counties to the south by 1999 (Gilliam et al., 2020). Additional county records from 2000 to 2020 across Illinois may, in part, represent increased effort to detect the tick, rather than true range expansion.

### 9.5. Eastward expansion from Illinois to Indiana

Two notable studies from Indiana published in the early to mid-1980s (Whitaker, 1982; Demaree, 1986) reported on examination of more than 20,000 ticks from a variety of sources within the state without finding any *I. scapularis* specimens, except from people or pets with out-of-state travel histories. In a review of the history of *I. scapularis* in Indiana, Pinger (2008) noted that through 1986 there was no direct evidence of this tick being established in the state. The first records of *I. scapularis* from Indiana came in 1987, when small numbers of adults were recovered from deer and a dog in counties in the far northwestern (LaPorte and Porter), north-central (Marshall and Wabash), and west-central (Parke) parts of the state, in most cases near the Illinois border for the ticks recovered from deer (Pinger and Glancy, 1989; USNTC, unpublished data). Additional records accumulated in 1989, with single adult *I. scapularis* taken from two humas and three dogs in counties (Madison, Marshall, Porter, and St. Joseph) in the northern part of Indiana (USNTC, unpublished data). This was followed up by examination of hunter-killed deer in 1990 from selected check stations located in the northern, west-central, and southern parts of the state (Pinger et al., 1991). *Ixodes scapularis* adults were collected from 10 deer in Newton County and one deer from Porter County in the far northwest, and from two deer in Vigo County in the west-central part of the state on the Illinois border. Additional deer surveys from 1991 to 1994 continued to recover *I. scapularis* from the northwestern part of the state, including in Jasper, Lake, LaPorte, Marshall, Newton, Porter, Pulaski, and Starke counties (Pinger et al., 1996). Moreover, a study in Jasper County in 1993 documented all life stages of *I. scapularis* by drag sampling, and larvae were recovered from rodents and shrews (Pinger et al., 1996). Most likely, *I. scapularis* invaded these areas in far western Indiana from eastern Illinois. Dennis et al. (1998) listed eight counties (Jasper, LaPorte, Newton, Porter, Pulaski, Starke, Vigo, and White) in western Indiana as having established populations of *I. scapularis* by 1996 (Fig. 3).

In contrast, no *I. scapularis* were detected on 170 deer from counties in the south or from nearly 300 deer in counties located in the central or eastern parts of far northern Indiana during the deer survey in 1990 (Pinger et al., 1991). Snider and Pinger (1995) then reported a similar result for deer surveys from 1991 to 1993. A later statewide deer survey conducted from 2005 to 2007 documented infestation with *I. scapularis* in all three years from the majority of counties in far western Indiana, and in one to three years from most counties in the central part of the state (Keefe, 2008). Counties without records of deer infested with *I. scapularis* were predominantly in the eastern part of the state. From 2004 to 2006, Diuk-Wasser et al. (2010) similarly recorded *I. scapularis* nymphs by drag sampling from sites in northwestern and west-central Indiana, near the Illinois border, but not from seven sites located in the central, eastern, or far southern parts of the state. It also should be noted that Pinger (2008) provided a map showing the first year of *I. scapularis* detection by county in Indiana. Most recently, established populations of *I. scapularis* were documented in seven additional counties, mainly in southern Indiana, based on collection of host-seeking ticks in 2018 and 2019 (Wojan et al., 2022; see also Fig. 3).

### 9.6. Expansion from western Pennsylvania into eastern Ohio

The first more recent record of *I. scapularis* from Ohio was a nymph, presumably taken from a human or pet, recovered in 1989 from Butler County in the far southwest (Wang et al., 2014). Dennis et al. (1998) listed three counties in far southwestern Ohio (Butler, Clermont, and Hamilton), one county in the far northwest (Williams), and one county in the east (Wayne) as having reports of *I. scapularis* by 1996, but none of them were classified as having an established tick population. Consistent with this, drag sampling conducted from 2004 to 2006 at 14 forested sites distributed across Ohio yielded no *I. scapularis* (Diuk-Wasser et al., 2010). Additional information from Ohio came from a passive tick surveillance program (soliciting ticks from the general public, health care providers, and local health departments) operated from 1983 to 2012 by the Ohio Department of Health (ODH); examination of hunter-killed deer for ticks from 1986 to 2011, and occasional rodent trapping; flagging for ticks at Lyme disease patient locations; and a focused effort in 2010 to collect ticks in Coshocton County in east-central Ohio (Wang et al., 2014). The number of *I. scapularis* submitted via the passive surveillance system was low through 2008, averaging less than 2 ticks per year and accounting for less than 1% of all ticks submitted. It is not clear to what extent these *I. scapularis* ticks may have been acquired while traveling out-of-state. Submissions of *I. scapularis* then increased dramatically from 2009 (15 ticks submitted) through 2011 (182 ticks) and 2012 (184 ticks), at which point *I. scapularis* accounted for 25% of all ticks submitted. Examination of hunter-killed deer in Ohio produced no *I. scapularis* from 2002 to 2008, based on 200–500 animals examined each year. Then, similar to the trend from the passive surveillance system, 29 *I. scapularis* adults were recovered from 200 examined deer in 2010 and this increased to 1830 *I. scapularis* adults from 560 examined deer in 2011. The vast majority of infested deer were from eastern Ohio, indicative of spread of *I. scapularis* from western Pennsylvania. Notably, the rapid increase in detection of *I. scapularis* from 2008 to 2011–2012 through passive tick submission or deer infestation is not attributable to increased surveillance. Additionally, active surveillance in Coshocton County in east-central Ohio in 2010 documented all life stages of *I. scapularis*. Recent Ohio Department of Health surveillance efforts show that *I. scapularis* ticks now are commonly collected in Ohio, with records from 50 of Ohio’s 88 counties in 2021 and from 66 counties in 2022 (ODH, 2023). Most counties meeting the criteria for *I. scapularis* being established in 2022 were located in eastern Ohio (Fig. 3).

### 9.7. Expansion from the western parts of Maryland and Pennsylvania into northern West Virginia

The USNTC includes one record of *I. scapularis* from 1991 in West Virginia: four adults taken from dog(s) in Jefferson County in the far eastern part of the state, bordering on Maryland and Virginia (USNTC, unpublished data). Dennis et al. (1998) listed only two counties (Berkeley and Jefferson) in far northeastern West Virginia as having established populations of *I. scapularis* by 1996. These counties border on Maryland to the north and are also very close to Pennsylvania. Increasing Lyme disease cases in West Virginia from 2014 to 2020 (CDC 2023c), primarily in the far northeastern part of the state including Berkeley and Jefferson counties, indicate proliferation of previously established *I. scapularis* populations in these two counties and spread to and proliferation in nearby counties. This was supported by a study on infestation of cats and dogs by *I. scapularis* in West Virginia from 2014 to 2016, with the majority of ticks recovered from counties in northern part of the state (Hendricks et al., 2017). It seems likely that northern populations of *I. scapularis* spread from Maryland and Pennsylvania into northern West Virginia. By 2022, *I. scapularis* was considered established in 40 counties spread across West Virginia but concentrated in the north (CDC, 2023b; Fig. 3).

### 9.8. Records from Missouri, Kentucky, and Tennessee

There are records of *I. scapularis* from counties in southern Missouri in the 1940s (Cooley and Kohls, 1945; USNTC, unpublished data), in the 1980s ( USNTC, unpublished data), from 1993 to 1996 (Kollars et al., 1997, 1999), and from 2004 to 2006 (Diuk-Wasser et al., 2010). The *I. scapularis* populations in southern Missouri most likely represented the northern edge for southern populations of this species; in a study including the seasonal peak activity periods for both nymphs and adults, the vast majority (>99%) of ticks collected by drag sampling were adults (Kollars et al., 1999). Diuk-Wasser et al. (2010) also sampled five sites in northern Missouri from 2004 to 2006, but without collecting any *I. scapularis* nymphs. However, from 2007 to 2015, *I. scapularis* ticks were consistently collected from Adair County in northeastern Missouri (Petry et al., 2010; Hahn and Foŕe, 2019). Intriguingly, all life stages of *I. scapularis* were collected by drag sampling and CO_2_-trapping in Adair County, raising the question of whether this may be a northern population of *I. scapularis*, perhaps having spread into northeastern Missouri from western Illinois.

County records for *I. scapularis* are scarce from Kentucky and Tennessee until recently: the tick was taken in 1911 from livestock in Fentress County in north-central Tennessee (USNTC, unpublished data); in 1990 from a dog in Ballard County in far western Kentucky, on the border with Illinois and Missouri (USNTC, unpublished data); and it was only listed by Dennis et al. (1998) as established in a single county (Shelby) in far southwestern Tennessee, and as reported in two counties in far western Kentucky (Ballard and Christian) and six counties (Bedford, Fentress, Lake, Marion, Rutherford, and Scott) across Tennessee. Studies over the last decade on *I. scapularis* in Tennessee and Kentucky, focusing on collection of adults from hunter-killed deer or via drag sampling, have generated new information on where in these states the tick occurs. Established populations of *I. scapularis* were documented from most counties in Tennessee (Rosen et al., 2012; Fritzen et al., 2014; Mays et al., 2014; Hickling et al., 2018) and counties across Kentucky (Lockwood et al., 2018; Pasternak and Palli, 2022).

## 10. Geographic distribution of *I. scapularis* records in the Southeast region (Alabama, Florida, Georgia, North Carolina, South Carolina, and Virginia), 1921 to present

The text below presents overviews of the geographic distribution of *I. scapularis* records in the Southeast region from 1921 to 1950 (Section 10.1), 1951 to 1975 (Section 10.2), and 1976 to present (Section 10.3), followed by a description of northern populations of *I. scapularis* from the Northeast moving into Virginia and North Carolina, where previously only southern populations of this species occurred (Sections 10.4–10.5; see also Fig. 4). Out of the 572 counties in the Southeast region, the cumulative number of counties with collection records of *I. scapularis* rose from 40 (7% of all counties) by 1950 and 66 (12%) by 1975 to 225 (39%) by 1996, 348 (61%) by 2015, and 397 (69%) by 2022 (Fig. 5).

### 10.1. Overview of collection records from 1921 to 1950

The period from 1921 to 1950 produced collections of *I. scapularis* from 16 counties spread across Florida: Alachua, Bay, Bradford, Duval, Franklin, Hardee, Highlands, Hillsborough, Lake, Marion, Osceola, Palm Beach, Putnam, Sarasota, Seminole, and Volusia (Hixson, 1941; Travis, 1941; Cooley and Kohls, 1945; Carpenter et al., 1946; Worth, 1950; Rogers, 1953; Persing et al., 1990; USNTC, unpublished data). Additional collection records came from nine counties in Georgia (Bryan, Burke, Camden, Chatham, Effingham, Grady, Liberty, Lowndes, and Thomas), two counties in Alabama (Baldwin and Sumter), and primarily coastal counties in North Carolina (Beaufort and Onslow), South Carolina (Allendale, Anderson, Berkeley, Charleston, Georgetown, and Horry) and Virginia (Richmond) (Cooley and Kohls, 1945; Carpenter et al., 1946; Pratt and McCauley, 1948; Morlan, 1952; Sonenshine et al., 1965; Wilson and Baker, 1972; Persing et al., 1990; USNTC, unpublished data). Most individual collections consisted of small numbers (<10) of adult ticks, taken from humans, domestic animals, and a wide variety of wildlife, or recovered by drag sampling. A few collections included larger numbers (hundreds) of larval or nymphal ticks, taken from lizards or various mammals (Hixson, 1941; Rogers, 1953).

### 10.2. Overview of collection records from 1951 to 1975

The period from 1951 to 1975 produced records of *I. scapularis* taken from a variety of hosts. Collections were made in six counties in Florida, including Alachua, Glades, Henry, Highlands, Indian River, and Polk (Rogers, 1953; Bryant et al., 1969; Wilson and Kale, 1972; USNTC, unpublished data); 14 counties in Georgia, including Brantley, Brooks, Bryan, Bulloch, Burke, Chatham, Cook, Grady, Indianola, Lowndes, McIntosh, Pierce, Ridgeville, and Terrell (Lund et al., 1962; Wilson and Baker, 1972; USNTC, unpublished data); and seven counties in Alabama, including Barbour, Bibb, Clarke, Colbert, Escambia, Pickens, and Winston (Cooney and Hayes, 1972). Additional *I. scapularis* records came from Brunswick and Onslow counties in North Carolina (Tibbetts, 1953; USNTC, unpublished data), and the independent city of Newport News as well as Augusta, James City, Nansemond, and Rockingham counties in Virginia (Sonenshine et al., 1965; Sonenshine and Stout, 1971; Heck, 1972; Sonenshine, 1979; USNTC, unpublished data). Records only reported at state level included *I. scapularis* taken from white-tailed deer in Alabama, Florida, Georgia, North Carolina, South Carolina, and Virginia from 1963 to 1970 (Kellogg et al., 1971) and from human, dog(s), and lizard(s) in South Carolina (Burgdorfer et al., 1975; Loving et al., 1978; USNTC, unpublished data). Finally, *I. scapularis* recorded in USDA annual tick surveys from 1963 to 1975 were predominantly from Florida with fewer ticks collected from Alabama, Georgia, North Carolina, South Carolina, and Virginia (USDA, Animal and Plant Health Inspection Service; unpublished data).

### 10.3. Overview of collection records from 1976 to present

The emergence of Lyme disease in the Northeast and Upper Midwest led to increased interest in *I. scapularis* also in the Southeast region. However, consistently low numbers of cases of Lyme disease in the Southeast region up to 2000 (CDC, 2023c) resulted in tick surveillance and research efforts focusing primarily on other tick species of greater regional public health concern, such as *D. variabilis* and *A. americanum* (Childs and Paddock, 2003; Paddock and Yabsley, 2007; Stromdahl and Hickling, 2012). Most states in the region (Alabama, Florida, Georgia, and South Carolina) still report low numbers of annual Lyme disease cases but the northernmost state, Virginia, experienced an increase in Lyme disease cases after 2000 and the same trend occurred for the neighboring state to the south, North Carolina, starting around 2012 (CDC, 2023c). These states are therefore also addressed separately in Sections 10.4 and 10.5.

Dennis et al. (1998) listed counties with established populations of *I. scapularis* from all states in the Southeast region by 1996: Alabama (25 counties spread across the state), Florida (35 counties spread across the state), Georgia (23 counties spread across the southern and central parts of the state), North Carolina (seven counties in the eastern part of the state), South Carolina (13 counties spread across the state), and Virginia (eight counties in the eastern part of the state). From 2004 to 2006, drag sampling focusing on the nymphal stage of *I. scapularis* was conducted across the Southeast region (Diuk-Wasser et al., 2010). Low numbers of nymphs were recovered from some sites in the eastern parts of Georgia, South Carolina, North Carolina, and Virginia, but the majority of sites across the region did not yield any nymphs. This is not surprising because, with the exception of the western parts of Virginia and North Carolina in recent years (see Sections 10.4–10.5), drag sampling has primarily yielded the adult stage of *I. scapularis* in the Southeast (Lavender and Oliver, 1996; Clark et al., 1998; Cilek and Olson, 2000; Maggi et al., 2010; Glass et al., 2019; Morris et al., 2022) and the vast majority of human encounters has been with adult ticks (Felz et al., 1996; Merten and Durden, 2000). Additional information on the geographic distribution of *I. scapularis* was published from Alabama (Kerr et al., 2022a,b), Florida (Forrester et al., 1996; Cilek and Olson, 2000; Durden et al., 2000; Allan et al., 2001; Fang et al., 2002; Burroughs et al., 2016; Hertz et al., 2017; Glass et al., 2019, 2021; Kessler et al., 2019), Georgia (Durden et al., 2002; Fang et al., 2002; Wells et al., 2004; Wedincamp and Durden, 2016), North Carolina (Apperson et al., 1990, 1993; Levine et al., 1997; Maggi et al., 2010; Barbarin et al., 2020), South Carolina (Barton et al., 1992; Clark et al., 1998, 2001; Fang et al., 2002), and Virginia (Sonenshine et al., 1995; Casteel and Sonenshine, 1996; Tanner et al., 2010; Brinkerhoff et al., 2014; Herrin et al., 2014; Kelly et al., 2014; Nadolny et al., 2014; Jackson, 2020; Morris et al., 2022; Whitlow et al., 2022). These studies added numerous new county records for *I. scapularis* in all states in the Southeast. Additional records of *I. scapularis* in the USNTC for the Southeast region after 1975 are primarily from a limited number of counties in Florida and Georgia, although there also are substantial records from coastal counties in North Carolina and Virginia (USNTC, unpublished data). By 2022, *I. scapularis* was documented to be widely distributed across the Southeast region, albeit a smaller proportion of individual counties have records of the tick compared to the Upper Midwest and Northeast regions (CDC, 2023b; Fig. 5).

### 10.4. Southward expansion of northern population ticks into western Virginia

As noted in Sections 10.1–10.3, *I. scapularis* was repeatedly recorded from coastal Virginia through the 1900s, and Dennis et al. (1998) listed the tick as established in eight coastal counties by 1996 (Fig. 3). Diuk-Wasser et al. (2010) conducted drag sampling from 2004 to 2006 in 12 forested sites evenly distributed throughout the state: the highest density of *I. scapularis* nymphs was recorded in north coastal Virginia and nymphs were less abundant or absent from inland sites and southern coastal sites. Up to 2005, the majority of Lyme disease cases also were reported from northeastern Virginia (Brinkerhoff et al., 2014). The number of Lyme disease cases reported from Virginia then tripled from 2006 to 2007, driven by a growing number of Lyme disease cases reported from counties along the Appalachian Mountains in the western part of the state (Brinkerhoff et al., 2014). This geographical shift in Lyme disease case locations in Virginia has remained through the present.

*Ixodes scapularis* nymphs were collected by drag sampling in 2011 from five counties (Appomattox, Buckingham, Goochland, Nelson, and New Kent) in northern Virginia, with the greatest density of nymphs recorded from Nelson County, located along the base of the Appalachian Mountains in the northwestern part of the state (Brinkerhoff et al., 2014). Moreover, nymphs from two of the counties (Goochland and Nelson) were infected with *Bo. burgdorferi* s.s. Subsequent studies conducted from 2012 to 2020 in the Appalachian Mountain region, which extends from north to south through western Virginia, documented host-seeking *I. scapularis* nymphs, and nymphs or adults infected with *Bo. burgdorferi* s.s., from numerous counties (Bedford, Botetourt, Floyd, Giles, Montgomery, Pulaski, Roanoke, Rockbridge, and Wythe) located in the central western and southwestern parts of the state (Herrin et al., 2014; Jackson, 2020; Morris et al., 2022; Whitlow et al., 2022). Moreover, Morris et al. (2022) found that *I. scapularis* nymphs quested openly and were readily collected by drag sampling in southwestern Virginia but not in the southeastern part of the state during 2018 to 2019.

The findings described above, together with information from population genetic studies (Kelly et al., 2014; Jackson, 2020), indicate that the increase in Lyme disease cases in Virginia from 2007 onward resulted from invasion and establishment of northern populations of *I. scapularis* with a host-seeking behavior more likely to result in contact with humans compared to previously established southern populations of the tick. It seems likely that the northern populations of *I. scapularis* spread from Maryland into northern Virginia and then southward along the Appalachian Mountains. By 2022, *I. scapularis* was considered established in 68 counties spread across Virginia (CDC, 2023b; Fig. 3).

### 10.5. Continued southward expansion of northern population ticks into northwestern North Carolina

Lyme disease cases started to increase around 2012 in North Carolina, driven by counties located in the Appalachian Mountain region in the far west of the state (CDC, 2023c; NCDHHS, 2023). It is reasonable to assume this resulted from continued southward spread of northern populations of *I. scapularis* along the Appalachian Mountains from southwestern Virginia into northwestern North Carolina. Data on host-seeking *I. scapularis* are scarce from this part of North Carolina. However, a cluster of Lyme disease cases in 2019 among children at an outdoor wilderness camp in Buncombe County in far western North Carolina motivated an entomological investigation (Barbarin et al., 2020). A total of 37 host-seeking *I. scapularis* nymphs were collected by drag/flag sampling, and six ticks were found to be infected with *Bo. burgdorferi* s.s. Another recent study (Clark and Herman-Giddens, 2023) recorded host-seeking larval and nymphal *I. scapularis* from Ashe County in far northwestern North Carolina and documented infection with *Bo. burgdorferi* s.s. in *P. leucopus* mice. By 2022, *I. scapularis* was considered established in 59 counties spread across North Carolina, including numerous counties in the far western part of the state (CDC, 2023b; Fig. 3).

## 11. Geographic distribution of *I. scapularis* records in the South region (Arkansas, Kansas, Louisiana, Mississippi, Oklahoma, and Texas), 1921 to present

The text below presents overviews of the geographic distribution of *I. scapularis* records in the South region from 1921 to 1950 (Section 11.1), 1951 to 1975 (Section 11.2), and 1976 to present (Section 11.3). Out of the 657 counties in the South region, the cumulative number of counties with collection records of *I. scapularis* rose from 54 (8% of all counties) by 1950 and 69 (11%) by 1975 to 119 (18%) by 1996, 206 (31%) by 2015, and 320 (49%) by 2022 (Fig. 5).

### 11.1. Overview of collection records from 1921 to 1950

The period from 1921 to 1950 produced collections of *I. scapularis* (including the synonym *I. ozarkus*) from 20 counties spread across the eastern part of Texas, including Angelina, Aransas, Bexar, Bowie, Brazoria, Brazos, Comal, Colorado, Henderson, Kleberg, Lavaca, Llano, Menard, Palo Pinto, Real, Robertson, Shelby, Sutton, Val Verde, and Zavala (Brennan, 1945; Cooley and Kohls, 1945; Randolph and Eads, 1946; Eads, 1949; Eads and Menzies, 1950; Eads et al., 1956; USNTC, unpublished data); and 15 counties across Arkansas, including Boone, Calhoun, Carroll, Conway, Dallas, Fulton, Garland, Izard, Lawrence, Madison, Montgomery, Ouachita, Polk, Pulaski, and Washington (Cooley, 1944; Cooley and Kohls, 1945; McAllister et al., 2016; USNTC, unpublished data). Additional collections were reported from Copiah, Forrest, Jackson, Harrison, and Wilkinson counties in Mississippi; Comanche and Latimer counties in Oklahoma; Cherokee County in Kansas; and unspecified locations in Louisiana (Cooley and Kohls, 1945; Carpenter et al., 1946; Philip and White, 1955; White, 1955; USNTC, unpublished data). Most individual collections consisted of small numbers (<10) of adult ticks, taken from humans, domestic animals, and a wide variety of wildlife, or recovered by drag sampling. A few collections included nymphal ticks, taken from lizards or various mammals (Eads et al., 1956).

### 11.2. Overview of collection records from 1951 to 1975

The period from 1951 to 1975 produced records of *I. scapularis* from 13 counties in Texas, including Aransas, Kerr, Lamar, Real, Robertson, Rusk, San Patricio, Shelley, Smith, Taylor, Upshur, Uvalde, and Walker (Eads et al., 1956; Drummond, 1967; Samuel and Trainer, 1970). Additional *I. scapularis* records came from Jefferson, Madison, and Washington counties in Arkansas (Harris, 1959; Tugwell and Lancaster, 1962; McAllister et al., 2016; USNTC, unpublished data); Rapides County in Louisiana (USNTC, unpublished data); Adams and Bolivar counties in Mississippi (USNTC, unpublished data); and Muskogee and Cherokee counties in Oklahoma (Clymer et al., 1970; Semtner and Hair, 1975). Records only reported at state level included *I. scapularis* taken from white-tailed deer in Arkansas, Louisiana, Mississippi, and Oklahoma from 1963 to 1970 (Kellogg et al., 1971). Finally, *I. scapularis* were commonly recorded in USDA annual tick surveys from 1963 to 1975 in Arkansas, Louisiana, Mississippi, Oklahoma, and Texas (USDA, Animal and Plant Health Inspection Service; unpublished data).

### 11.3. Overview of collection records from 1976 to present

The emergence of Lyme disease in the Northeast and Upper Midwest led to increased interest in *I. scapularis* also in the South region. However, consistently low numbers of cases of Lyme disease in the South region up to present time (CDC, 2023c) has resulted in tick surveillance and research efforts focusing primarily on other tick species of greater regional public health concern, such as *D. variabilis, A. americanum*, and the Gulf Coast tick, *Amblyomma maculatum* (Childs and Paddock, 2003; Paddock and Yabsley, 2007; Stromdahl and Hickling, 2012; Paddock and Goddard, 2015). Dennis et al. (1998) listed counties with established populations of *I. scapularis* from all states in the South region by 1996: Arkansas (9 counties spread across the state), Kansas (Douglas County in the eastern part of the state), Louisiana (12 counties primarily in the northern part of the state), Mississippi (10 counties spread across the state), Oklahoma (Cherokee, Le Flore, and McClain counties in the eastern part of the state), and Texas (24 counties in the eastern part of the state) (Fig. 3). From 2004 to 2006, drag sampling focused on the nymphal stage of *I. scapularis* was conducted across the South region, but with minimal success (Diuk-Wasser et al., 2010). This is not surprising because drag sampling has primarily yielded the adult stage of *I. scapularis* across the region (Mackay and Foil, 2005; Goltz and Goddard, 2013; Leydet and Liang, 2014; Mitcham et al., 2017; Tietjen et al., 2019, 2020; Raghavan et al., 2021; Spare et al., 2021) and the vast majority of human encounters has been with adult ticks (Merten and Durden, 2000; Goddard, 2002; Williamson et al., 2010; Dowling et al., 2022). More recent information on the geographic distribution of *I. scapularis* was published from Arkansas (Trout and Steelman, 2010; Dowling et al., 2022), Kansas (Coleman, 2013; Raghavan et al., 2021; Spare et al., 2021), Louisiana (Mackay and Foil, 2005; Leydet and Liang, 2013, 2014), Mississippi (Demarais et al., 1987; Goddard, 2002; Goddard et al., 2003; Godard and Piesman, 2006; Goltz and Goddard, 2013), Oklahoma (Koch and Dunn, 1980; Garvin et al., 2015; Mitcham et al., 2017; Small et al., 2019), and Texas (Williamson et al., 2010; Sanders et al., 2013; Santelises, 2013; Feria-Arroyo et al., 2014; Tietjen et al., 2019, 2020; Olafson et al., 2020; Mendoza, 2021). These studies added numerous new county records for *I. scapularis*, including in Kansas and Oklahoma. Additional records of *I. scapularis* in the USNTC are limited for the South region after 1975, including only roughly 120 specimens collected primarily from Arkansas, Kansas, Mississippi, and Oklahoma (USNTC, unpublished data). By 2022, *I. scapularis* was documented to be widely distributed across forested portions of the South region, and along riparian corridors extending into the open plains, albeit a smaller proportion of individual counties have records of the tick compared to the Upper Midwest and Northeast regions (CDC, 2023b; Fig. 5).

## 12. Conclusions

*Ixodes scapularis* appears to be reclaiming a historical range in the northern part of the eastern US. Surveillance data for this tick were limited prior to the 1980s, and national tick surveillance and reporting efforts were not standardized until 2018 (Eisen and Paddock, 2021), which limits our ability to track changes in the distribution of *I. scapularis* over time. Nonetheless, based on trends in the tick’s distribution and spread presented in this review, it appears that northern populations of *I. scapularis* persisted in refugia sites in Wisconsin in the Upper Midwest and in the coastal Northeast. Additional potential refugia or early reintroduction sites include the western Upper Peninsula of Michigan and far northwestern Pennsylvania bordering southwestern New York. Perhaps not coincidentally, the latter area is in close proximity to a known refugium site for *I. scapularis* in Long Point (Ontario, Canada).

Expansion of *I. scapularis* in all geographic directions from the main refugia sites appears to have been rapid from the 1970s to present. Records suggest expansion in the Northeast preceded expansion in the upper Midwest, which might be explained by earlier reforestation and repopulation of deer in the Northeast. Despite an earlier start in the Northeast, tick expansion in the Northeast and Upper Midwest occurred concurrently from the 1970s. These northern foci, and the persistent populations in the South and Southeast, likely seeded the current tick populations in the Ohio Valley from the north and south, respectively. Incursion into Virginia and North Carolina of northern population *I. scapularis* appears to have originated from the Northeast focus. The relative importance of different hosts, such as deer and birds, for the spread of *I. scapularis* to new areas remains unclear.

In general, there was a consistent and expected trend of early detection of the tick at low densities with abundance stabilizing over time resulting in gradients of high to low densities along the colonization pathways. Reports of Lyme disease cases generally followed similar trends as tick colonization, although in some areas, cases were reported prior to recognition of established tick populations; in those situations, the occurrence of disease cases often prompted tick surveillance efforts to confirm the local presence of infected vector ticks. It is quite likely we will continue to see increases in densities of host-seeking *I. scapularis* nymphs and human-tick encounter rates in areas that were recently colonized by northern populations of *I. scapularis* (e.g., the western and southern edges of the current known distribution for northern population ticks; Figs. 3, 4), with subsequent increases in incidence of *I. scapularis*-borne diseases.

Reforestation, repopulation of white-tailed deer, and changing climatic conditions likely all contributed to the changing geographic distribution of *I. scapularis*. However, the relative contributions of each factor at a national scale are currently unknown. Ongoing surveillance of ticks and tick-borne pathogens is essential to provide the data needed for studies that seek to evaluate the relative roles of land cover, tick hosts, and climate in explaining and predicting geographic expansion of ticks and tick-borne diseases.

## Data Availability

No data was used for the research described in the article.
